# Supplementary material for: Bio-inspired decision making in robot swarms under biases
Source: Nat Commun. 2026 Aug 14;17:9774. doi: 10.1038/s41467-026-76408-4 (PMC13572407; doi:10.1038/s41467-026-76408-4)
Supplement: Supplementary file 1 [file 41467_2026_76408_MOESM13_ESM.pdf]

nature portfolio

Peer Review File

Bio-inspired decision making in robot swarms under biases

Corresponding Author: Ms Raina Zakir

This file contains all reviewer reports in order by version, followed by all author rebuttals in order by version.

Version 0:

Reviewer comments:

Reviewer #1

(Remarks to the Author)
The best-of-n decision problem is crucial, both for understanding collective decision-making behaviors in biological swarms and for developing algorithms for swarm robots. This manuscript proposes a unified model to study collective decision-making under asocial biases, which can arise from multiple factors, with a focus on the effects of cross-inhibition. I consider this research meaningful, but when evaluated against NC's consistent high standards, the current manuscript requires revisions.

My main comments are as follows:

1. For NC's high-quality standards, the Introduction and Discussion sections are overly lengthy. While they contain rich information and insightful observations, their excessive length hinders readers from grasping the core arguments. A paper typically works best when focused on a single key issue; overexpansion of content undermines this goal, and a more balanced scope should be adopted. Additionally, the first three parts of the Results contain redundant descriptions and explanatory text. If the authors deem this content necessary, I suggest relocating it to the Supporting Information (SI).

2. In my view, there are too many figures in the manuscript; 5–6 figures would be appropriate. Some figures lack clarity, including overly small font sizes for axes and indistinct curves. Moreover, "ci"/"ds" and "CI"/"DS" are used interchangeably in the figures—consistency should be maintained. I highly appreciate NC's consistently high standards for figure presentation and would encourage the authors to revise and provide high-quality figures.

3. I recommend moving Figure 1 to the SI, as it serves little purpose in the main text. The labels "opinion A" and "opinion B" may be confused with the subfigure labels "A" and "B". It is suggested to use "stateA/B/U" instead, as "Committed to A/B" could be misleading. While the use of bubbles and envelopes to represent information transmission is creative and stylized, it is not straightforward; consider using arrows to directly indicate information flow and exchange. Furthermore, the 3D illustration using Kilobot robot images is not intuitive and does not help readers quickly grasp the authors' intent.

4. The authors discuss the best-of-n problem in the Abstract and Introduction, but the actual research focuses on best-of-2 (two-choice) decision-making. Although binary decision-making is indeed a subset of best-of-n problems, it may lack generality. I would like to know whether the conclusions drawn are generalizable.

5. The title of the manuscript could be streamlined by removing the phrase "from stubborn robots, corrupted communication, and independent discovery," as the authors do not specifically investigate the differences between these three scenarios. While these are indeed causes of asocial biases, the authors unify them into a single model (lines 110–136), making them theoretically equivalent. Additionally, repeated mentions in various parts of the manuscript of examples such as "self-sourcing, asocial agents like zealots, or communication corruption" are redundant and unnecessary.

6. Another issue I wish to highlight is that the manuscript (starting from line 200) reports phenomena observed in simulation analyses without deeply exploring the underlying mechanisms, nor deriving more profound insights. This depth of analysis is essential.

7. The manuscript uses numerous symbols, which can easily confuse readers. I suggest compiling a table to centrally introduce these symbols for quick reference, thereby improving readability.

8. I noticed some inconsistent statements and would like to confirm with the authors: Were experiments conducted using real physical robots, or only simulations? The Introduction mentions "stochastic swarm robotics simulations," and Figure 3B does not appear to show physical robots, yet the Methods section claims that both robot experiments and simulations were performed. Consistency across sections is necessary.

9. Regarding the description of robot behavior in the Methods section, I believe using a finite state machine or pseudocode would be more concise and clear than pure textual description.

10. The first two sentences of the Abstract (lines 1–4) provide general background on swarm robotics and could be condensed or removed.

(Remarks on code availability)

Reviewer #2

(Remarks to the Author)
This manuscript compares two decision-making mechanisms, i.e., direct-switch and cross-inhibition, in a best-of-two problem problem under asocial dynamics within swarm systems. The results indicate that cross-inhibition, a biologically inspired mechanism, enables robust decision-making despite the presence of asocial dynamics. While the manuscript presents interesting results, its current organization requires readers to frequently jump between sections because key contextual descriptions appear later in the text. Reorganizing the manuscript so that essential descriptions appear earlier would enhance clarity and readability.

The manuscript focuses solely on conditions with two options. However, typical decision-making problems in natural or robotic systems often involve more than two options. Would the authors expect similar results in such cases? Any discussion or insights regarding the generalization of the proposed models to scenarios with a larger number of options would strengthen the manuscript.

I had difficulty interpreting Figure 8. I assume that CI and DS represent cross-inhibition and direct-switch, but this should be explicitly stated. I assume that cross-inhibition uses type 2 in Figure 8 (A), but this is never explained in the caption. In the third two rows in Figure 8 (A), the manuscript states that "Direct-switch shows higher accuracy in the bottom-left region," but the figure itself appears to indicate higher accuracy in the right region. Higher accuracy compared to what baseline? In addition, I did not understand how the results shown in panels (B) and (C) agree with those in panel (A). It would be helpful if the authors could indicate which parts of Figure 8 (A) correspond to (B) and (C).

(Remarks on code availability)
I cannot find the Github page. Maybe, the authors are planning to make it public after the manuscript is accepted.

Reviewer #3

(Remarks to the Author)
This manuscript investigates how minimalistic robot swarms make collective decisions under various forms of biased or asocial dynamics -- stubborn agents (zealots), corrupted communication, and independent environmental discovery -- by comparing the direct-switch mechanism (weighted voter) with the bio-inspired cross-inhibition mechanism. The authors present a multiscale modelling framework comprising a simplified mean-field ODE system, a more detailed robot-specific ODE formulation, master-equation formulations for probabilistic simulations using the Gillespie algorithm, and extensive Kilobot-based swarm simulations. A key conceptual contribution is the elegant mathematical unification of different bias sources through a single set of parameters (η, η_a, η_b), showing that zealots, MITM-style message corruption, and self-sourcing can all be treated as manifestations of the same underlying noisy update process. This theoretical abstraction is appealing and enables a systematic exploration of how bias interacts with decision dynamics. The results, which demonstrate that cross-inhibition achieves faster, more cohesive, and more accurate decisions across a wider range of biased conditions than direct-switch, are consistent across the different modelling layers and clearly visualised through bifurcation diagrams, stationary probability distribution comparisons, and simulation heatmaps.

However, while the multiscale methodology is solid and the internal consistency between the models and simulations is convincing, several issues limit the strength of the conclusions. First, many of the qualitative findings such as the susceptibility of direct-switch to deadlock under noise, the robustness of cross-inhibition, and the improvement in accuracy due to moderate noise are conceptually in line with existing literature in swarm robotics, distributed decision-making, and biological collective behaviour. The manuscript extends these insights rather than fundamentally revising them. Second, the generality of the proposed equivalence between different forms of asocial dynamics is somewhat overstated. The algebraic mapping is valid at the mean-field macro level, but it suppresses behavioural asymmetries: zealots are persistent state-holders, MITM attacks act per-message, and self-sourcing is state-dependent and embodiment-dependent. These distinctions may matter significantly in practice, particularly for finite or spatially structured systems, and should be more explicitly acknowledged. Third, some model–simulation mismatches, noted by the authors themselves (e.g., differences between simple vs. robot-specific ODE curves in Fig. 4), suggest that the simpler models only provide qualitative predictions, which in turn tempers the claim of broad applicability across biological systems.

The most significant limitation, however, lies in the practical feasibility of implementation and the lack of physical robot experiments, which strongly affects the generalisability of the results. Nearly all algorithmic assumptions -- single-neighbour polling, reliable and isolated message reception, unambiguous detection of conflicting opinions, uniform environmental sampling, and fixed-duration behavioural phases -- are idealised and difficult to satisfy with real minimal robots such as Kilobots. Real systems experience overlapping broadcasts, packet collisions, line-of-sight effects, motor noise, spatial clustering, and heterogeneous sensing, none of which are reflected in the modelling. Additionally, the probability of self-sourcing is treated as a controllable parameter, yet in real embodied systems it emerges from movement, occlusion, and sensing dynamics, making it neither uniform nor tunable. Even zealots, as modelled, represent an idealised failure mode: real malfunctioning robots fail multimodally, often altering mobility or communication range rather than merely freezing their opinion. Without hardware experiments validating at least the qualitative trends, it remains unclear whether the observed superiority of cross-inhibition under noisy conditions would persist in a physically embodied swarm.

Despite these limitations, the manuscript is clear, well written, and technically rigorous, and the unified bias framework is a genuine conceptual contribution. The multiscale alignment between ODEs, stochastic simulations, and robot simulations is commendable. However, the authors should temper their broader claims about biological generality and real-world applicability, and more explicitly discuss the constraints imposed by the idealised assumptions embedded in their modelling framework. Addressing these limitations through physical robot validation and a deeper analysis of how embodied noise and spatial structure might shift the stability landscape would strengthen the paper and its suitability for Nature Communications.

(Remarks on code availability)
The code is not available at https://github.com/rainazakir/asocialdynamics; am receiving a 404 error.

Version 1:

Reviewer comments:

Reviewer #2

(Remarks to the Author)
The authors have adequately addressed my previous comments. However, I have one minor comment regarding the structure of the manuscript. The figures should be cited in numerical order according to their appearance in the text. Currently, Figure 2 is referenced before Figure 1. It may not be necessary to refer to Figure 2 in the Introduction section.

(Remarks on code availability)
I attempted to install argos3-kilobot, but encountered a CMake version error and was unable to complete the setup. Therefore, I could not fully review or execute the code. However, I noticed that the README.md should be revised to match the directory names in the GitHub repository. Additionally, providing a README.md file for each simulation directory would improve clarity and make it easier to understand how to run the code.

Reviewer #3

(Remarks to the Author)
I thank the authors for their detailed response to the reviewers and for the substantial revisions made to the manuscript. In my view, the revised version is considerably stronger than the original submission. The authors have addressed the main concerns raised in my previous review in a serious and constructive manner.

My earlier concerns were mainly around the level of novelty, the breadth of some of the claims, the reliance on idealised models and simulations, and the lack of physical robot validation. The revised manuscript now makes the contribution clearer. I still do not see the paper as proposing a fundamentally new decision-making algorithm, since direct-switch and cross-inhibition are well-established mechanisms. However, the value of the paper is now better framed as a unified and systematic analysis of how different forms of asocial dynamics and bias affect collective decision-making in robot swarms. This is a reasonable and useful contribution, particularly because the analysis connects mean-field models, stochastic simulations, robot simulations, multi-option decision-making, and physical robot demonstrations.

The addition of real-robot experiments is an important improvement. These experiments are necessarily limited in scope, but they help address my concern that the original manuscript relied too heavily on idealised assumptions and simulation results. I think the authors are right to present these experiments as qualitative validation rather than exhaustive hardware validation. The agreement between the physical experiments, robot simulations, and models strengthens the paper.

I am also satisfied with the authors' response regarding the equivalence between different forms of asocial dynamics. The revised manuscript is clearer that this equivalence is primarily macroscopic and model-level, and that implementation-specific differences may still matter in finite, spatially structured, or physically embodied swarms. This caveat is important and should remain visible in the final version.

The extension to decision-making with more than two options also improves the manuscript. It does not completely remove the fact that much of the paper is still built around the binary case, but it does show that the main qualitative trends are not restricted to best-of-2 decisions. This is sufficient for the current paper.

I have only a few remaining minor comments.

1. The authors should continue to be careful not to overstate the physical validation. The experiments support the qualitative trends, but they do not validate the full parameter space or all forms of asocial dynamics considered in the modelling framework.

2. Some of the broader claims about biological systems and general decentralised decision-making systems should remain carefully phrased. The manuscript is strongest when it speaks about robot swarms and model systems. The biological analogies are useful, but should not be presented as direct validation of the model.

3. The speed comparison remains somewhat dependent on how undecided runs are treated. I do not think this requires new experiments, but the manuscript would benefit from a short clarification that the interpretation of decision speed is tied to the quorum-based stopping criterion and to how non-decisions are handled.

Overall, I think the authors have addressed the major issues raised in the previous round. The revised manuscript is clearer, better supported, and more appropriately caveated.

(Remarks on code availability)
I have not performed a full code review and did not install or run the code. I therefore cannot independently assess the reproducibility of the results from the provided repository. I did inspect the repository briefly. It appears to contain code for the Gillespie simulations, robot simulations, plotting, Mathematica analyses, and Kilobot-related experiments. The repository also includes a README with some installation and execution instructions, including dependencies and scripts for generating simulation data and figures. This is useful, but without running the code I cannot comment on whether the instructions are sufficient in practice, whether all dependencies are correctly specified, or whether the paper's figures can be reproduced end-to-end. My assessment of the manuscript is therefore based on the paper, supplementary material, and response to reviewers, rather than on an independent verification of the code.

Open Access This Peer Review File is licensed under a Creative Commons Attribution 4.0 International License, which permits use, sharing, adaptation, distribution and reproduction in any medium or format, as long as you give appropriate credit to the original author(s) and the source, provide a link to the Creative Commons license, and indicate if changes were made.
In cases where reviewers are anonymous, credit should be given to 'Anonymous Referee' and the source.
The images or other third party material in this Peer Review File are included in the article's Creative Commons license, unless indicated otherwise in a credit line to the material. If material is not included in the article's Creative Commons license and your intended use is not permitted by statutory regulation or exceeds the permitted use, you will need to obtain permission directly from the copyright holder.
To view a copy of this license, visit https://creativecommons.org/licenses/by/4.0/

Responses to Reviewers

Manuscript ID : NCOMMS-25-73624

Submitted to Nature Communications

Bio-inspired decision making in robot swarms under biases

Raina Zakir[1,⋆] Timoteo Carletti[2] Marco Dorigo[1] Andreagiovanni Reina[3,4,∗]

[1]IRIDIA, Université Libre de Bruxelles, Brussels, Belgium,
[2]Department of Mathematics and Namur Institute for Complex Systems, naXys, University of Namur, Namur, Belgium
[3]Centre for the Advanced Study of Collective Behaviour, Universität Konstanz, Konstanz, Germany,
[4]Department of Collective Behaviour, Max Planck Institute of Animal Behavior, Konstanz, Germany
⋆ corresponding author, raina.zakir@ulb.be, andreagiovanni.reina@uni-konstanz.de

Dear Editor,

Below, we provide a complete list of the reviewers' comments along with our responses. To facilitate the review process, we also submit a version of the manuscript with all changes highlighted in red and blue. These highlights were generated automatically using latexdiff and, therefore, include all modifications, including minor ones. Before detailing the revisions made to the original version of the manuscript, we would like to thank the reviewers for their constructive and insightful comments. We have carefully addressed all the points raised and have substantially revised the manuscript. Where appropriate, excerpts from the manuscript are shown in blue to clearly distinguish them from our responses. In cases where we believe no modification was necessary, we provide a clear and reasoned justification for our decision.

Reviewer 1

The best-of-n decision problem is crucial, both for understanding collective decision-making behaviors in biological swarms and for developing algorithms for swarm robots. This manuscript proposes a unified model to study collective decision-making under asocial biases, which can arise from multiple factors, with a focus on the effects of cross-inhibition. I consider this research meaningful, but when evaluated against NC's consistent high standards, the current manuscript requires revisions.

1 - **For NC's high-quality standards, the Introduction and Discussion sections are overly lengthy. While they contain rich information and insightful observations, their excessive length hinders readers from grasping the core arguments. A paper typically works best when focused on a single key issue; overexpansion of content undermines this goal, and a more balanced scope should be adopted. Additionally, the first three parts of the Results contain redundant descriptions and explanatory text. If the authors deem this content necessary, I suggest relocating it to the Supporting Information (SI).**

Response - We agree with the reviewer that these sections can be consolidated. Accordingly, we have revised and shortened the Introduction by removing extended background on robot swarms and general collective decision-making. We have also substantially reduced the discussion of prior analyses of asocial dynamics and removed overly detailed explanations of the ODE models used to derive our results. The revised version focuses more directly on the specific contributions of this work. In addition, we have edited the text throughout to simplify and shorten excessively long sentences where possible.

We have kept the Discussion section largely unchanged, except for trimming redundant descriptions of the three asocial dynamics considered in this work. We believe the Discussion remains comprehensive and effectively conveys the relevance of the trade-offs highlighted by the two decision-making models we analyse. It also allows us to generalise our findings to other systems, relate our results to phenomena observed in real-world contexts, and clarify how the models may be beneficial depending on the scenario under consideration, thereby helping to guide their practical application.

We have revised the first three sections of Results. In particular, we removed the subsection "Equivalence of asocial dynamics" and relocated relevant parts to the Methods, where they fit more appropriately. In its place, we have added a new section entitled "Collective Decision-Making with more than two options," following the reviewer's suggestion to extend the discussion beyond binary choices.

We agree with the reviewer that the Results should remain focused and concise. At the same time, we consider the sections "Collective perception with a minimalistic robot swarm" and "Multiscale modelling: from macroscopic population dynamics to robot swarm experiments" essential for conveying the main contributions of our work. Following the reviewers' advice, we have substantially streamlined both sections. In "Collective perception with a minimalistic robot swarm," we removed redundant material already covered elsewhere and revised the text to be more concise and direct. We also improved the clarity of "Multiscale modelling: from macroscopic population dynamics to robot swarm experiments". To strengthen the multiscale narrative, we now briefly discuss the additional real-robot experiments included in the revision, making this section more complete with respect to the multiscale analysis. Finally, to improve accessibility, we reorganised the section "Fast, cohesive, accurate, and scalable collective decisions" by moving some background details from the introduction. The section now begins with a brief overview that highlights the importance of the metrics analysed

2 - **In my view, there are too many figures in the manuscript; 5–6 figures would be appropriate. Some figures lack clarity, including overly small font sizes for axes and indistinct curves. Moreover, "ci"/"ds" and "CI"/"DS" are used interchangeably in the figures—consistency should be maintained. I highly appreciate NC's consistently high standards for figure presentation and would encourage the authors to revise and provide high-quality figures.**

Response - We agree with the reviewer that the number of figures can be reduced and consolidated. To address this point, we have made the following changes:

(i) Former Figure 5 (stationary probability distribution analysis) has been moved to the Supplementary Material (now Figure SF9).

(ii) Redundant illustrations of the decision-making models have been removed from Figure 1. In addition, the portion of Figure 2 showing the schematic of the robot opinion update has been merged into Figure 1. As a result, Figure 2 now comprises the finite-state-machine diagram, the decision-making models, strip

plots of the quality estimates, the experimental arena, and the chemical-reaction-network representation. Consequently, the original Figures 1–3 have been consolidated into two figures.

(iii) Figures 6 and 8 have been combined (now Figure 5 in the revised manuscript).

(iv) To address the reviewer's fourth comment regarding the generalisation of the results to $n > 2$, we have added a new figure presenting the relevant analysis (Figure 7 in the revised manuscript).

We have also improved the graphical quality of the figures, particularly Figures 1 and 2. Axis font sizes have been increased and curve visibility enhanced, notably in Figure 6 (previously Figure 9). In addition, we ensured consistent terminology throughout (using only "CI" and "DS").

As a result of these changes, the number of figures in the main text has been reduced to seven, while the overall depth of the analysis has been improved.

3 - **I recommend moving Figure 1 to the SI, as it serves little purpose in the main text. The labels "opinion A" and "opinion B" may be confused with the subfigure labels "A" and "B". It is suggested to use "stateA/B/U" instead, as "Committed to A/B" could be misleading. While the use of bubbles and envelopes to represent information transmission is creative and stylized, it is not straightforward; consider using arrows to directly indicate information flow and exchange. Furthermore, the 3D illustration using Kilobot robot images is not intuitive and does not help readers quickly grasp the authors' intent.**

Response - We respectfully prefer to retain Figure 1 in the main manuscript rather than moving it to the Supplementary Material, as it plays an important conceptual role. The figure sets the tone of the paper and provides a consolidated visual overview of our approach. In particular, it illustrates the equivalence among the three modes of asocial dynamics, allowing readers to grasp the study's core idea at a glance and supporting the manuscript's overall narrative structure.

Following the reviewer's suggestion, we have nevertheless revised the figure to improve its clarity. We have introduced arrows to indicate the exchange of messages between robots and we also simplified the graphical elements to help distinguishing between the message sender and the message receiver. As our analysis, particularly the robot simulations and experimental demonstrations, was performed using Kilobots, we have kept the Kilobot representation in the illustration to ensure consistency with the experimental platform described in the paper.

In addition, to further enhance readability, we have replaced the labels "Committed to A/B" with "state A/B" and "Uncommitted" (in Figure 2 A-B), as suggested by the reviewer.

4 - **The authors discuss the best-of-n problem in the Abstract and Introduction, but the actual research focuses on best-of-2 (two-choice) decision-making. Although binary decision-making is indeed a subset of best-of-n problems, it may lack generality. I would like to know whether the conclusions drawn are generalizable.**

Response - We have extended the study by adding a new section, "Collective decision-making with more than two options", and a corresponding Figure 7. This addition analyses the impact of increasing asocial dynamics for $n = 3$ and $n = 5$ using both ODE models and robot simulations, across a range of quality ratios. The qualitative trends remain consistent with the $n = 2$ case: cross-inhibition continues to break symmetry and maintain cohesive decisions, whereas direct-switch increasingly leads to weak majorities or deadlock as n increases.

We would also like to highlight that, in both natural and artificial systems, decision problems with $n > 2$ alternatives are often effectively reduced to a sequence of binary ($n = 2$) decisions. This abstraction has been reported in several empirical and modelling studies ([Sridhar et al., PNAS 2021]; [Oddi et al., ANTS 2022]; [Lee et al., ANTS 2018]). In addition, other performance metrics—such as decision speed—could in principle be analysed for the $n > 2$ case. However, rather than duplicating well-established results, we refer to the broader body of work on collective decision-making showing that decisions typically become slower as the number of alternatives increases (e.g., Hick's law). In the Discussion section, we briefly mention this as follows:

While natural and artificial systems often approximate multi-alternative decisions as sequences of binary comparisons, we also analyse best-of-n dynamics directly for $n > 2$ and recover the same qualitative trends as in the binary case. More broadly, decision-making is known to slow as the number of alternatives increases (Hick–Hyman's law), making mechanisms that reliably break symmetry and maintain cohesion increasingly relevant in multi-option settings.

To examine the impact of asocial dynamic bias, we have also included Figure SF12 in the Supplementary Material, which reports the effect of the self-sourcing bias of the inferior option B on the selection of the best option for $n > 2$. As in the $n = 2$ case, similar dynamics are observed: the direct-switch mechanism establishes a majority for the best option only within a limited range of η_b, and this majority decreases as the number of options increases. Cross-inhibition, on the other hand, does not lead to deadlock, but its propensity to select the inferior-quality option increases in more difficult decision problems (i.e., for smaller quality ratios).

5 - **The title of the manuscript could be streamlined by removing the phrase "from stubborn robots, corrupted communication, and independent discovery," as the authors do not specifically investigate the differences between these three scenarios. While these are indeed causes of asocial biases, the authors unify them into a single model (lines 110–136), making them theoretically equivalent. Additionally, repeated mentions in various parts of the manuscript of examples such as "self-sourcing, asocial agents like zealots, or communication corruption" are redundant and unnecessary.**

Response - As suggested by the reviewer, we have shortened the title to: Bio-inspired decision making in robot swarms under biases.

Following the reviewer's comment, we have removed overly detailed and repetitive discussion of the three forms of asocial bias from the Introduction. We now refer to these only briefly when introducing the topic in the Introduction (lines 58-60), at the beginning of Results section (lines 90-92), in the Discussion (lines 255-257; 287-289), and in Figure 1.

6 - **Another issue I wish to highlight is that the manuscript (starting from line 200) reports phenomena observed in simulation analyses without deeply exploring the underlying mechanisms, nor deriving more profound insights. This depth of analysis is essential.**

Response - We thank the reviewer for the feedback.

The analysis of decision speed, cohesion, and accuracy (now introduced around line 164 in the revised manuscript) constitutes one of the central contributions of this work. We respectfully note that the Results section is structured to guide the reader through the observed phenomena, highlighting the key aspects of the data and figures that may not be immediately apparent without explicit explanation.

For example, in the direct-switch case, we show that even for relatively low η, the asocial bias η_a can offset quality differences and lead to decision deadlocks or weak majorities. The critical bias η_a^* (black line in the last row of Figure 5) marks the point at which the majority opinion shifts between options. Around this point, we observe in Figure 5 that the system exhibits poor cohesion. In contrast, even under strong asocial dynamics ($\eta \lesssim 0.4$ for noise type-1 and $\eta \lesssim 0.17$ for noise type-2), cross-inhibition breaks symmetry and drives the swarm toward a clear majority, regardless of q or η_a. This distinction is highlighted in the cohesion heatmaps of Figure 5.

As another example, we discuss how the direct-switch strategy, although it avoids selecting the inferior option when asocial dynamics are weak, is not stable because it is from transient fluctuations: the system hovers near an indecisive equilibrium, where small shifts in the majority can occasionally cross the quorum threshold Q (see the robot distribution in Figures 3 and 4). Because this equilibrium is biased toward the better option, such momentary majorities occur more frequently in its favour... resulting in highly variable decision times (see Supplementary Figure SF4).

The Discussion builds on these results by linking the observed patterns to their underlying mechanisms and outlining their broader implications.

7 - **The manuscript uses numerous symbols, which can easily confuse readers. I suggest compiling a table to centrally introduce these symbols for quick reference, thereby improving readability.**

Response - We thank the reviewer for this helpful suggestion. A comprehensive table summarising all symbols has been added to the Supplementary Material (Table SL1).

8 - **I noticed some inconsistent statements and would like to confirm with the authors: Were experiments conducted using real physical robots, or only simulations? The Introduction mentions "stochastic swarm robotics simulations," and Figure 3B does not appear to show physical robots, yet the Methods section claims that both robot experiments and simulations were performed. Consistency across sections is necessary.**

Response - We agree with the reviewer on the importance of maintaining consistent terminology. In the original submission, the experiments were conducted using robot simulations only. As we have now included real-robot experiments, we have revised the manuscript accordingly and clarified the terminology, distinguishing explicitly between simulations and physical experiments. The results of the real-robot demonstrations are now included in Figures 3 and 4 of the revised manuscript. The distinction between simulations and experiments is made explicit throughout the text (for example, lines 81, 161, 208, 246).

9 - Regarding the description of robot behavior in the Methods section, I believe using a finite state machine or pseudocode would be more concise and clear than pure textual description.

Response - We thank the reviewer for this helpful suggestion. A finite-state machine representation was already shown in Figure 3 (Figure 2C in the revised manuscript). To further clarify the implementation, we have added the corresponding pseudocode in the Supplementary Material (Algorithm SA1) and explicitly referenced it in the main manuscript (line 496).

10 - The first two sentences of the Abstract (lines 1–4) provide general background on swarm robotics and could be condensed or removed.

Response - We agree with the reviewer's comment. In the revised manuscript, we have made the text more concise and refocused it specifically on the problem of collective decision-making, rather than providing a general discussion of swarm robotics. Accordingly, we now state:

To operate autonomously, minimal robot swarms must make timely and reliable collective decisions despite noisy individual sensing and severe constraints on communication, computation, and memory. Achieving this capability could expand their use in applications such as healthcare, disaster response, and environmental monitoring.

Reviewer 2

1 - This manuscript compares two decision-making mechanisms, i.e., direct-switch and cross-inhibition, in a best-of-two problem problem under asocial dynamics within swarm systems. The results indicate that cross-inhibition, a biologically inspired mechanism, enables robust decision-making despite the presence of asocial dynamics. While the manuscript presents interesting results, its current organization requires readers to frequently jump between sections because key contextual descriptions appear later in the text. Reorganizing the manuscript so that essential descriptions appear earlier would enhance clarity and readability.

To do - We have revised the structure of the Introduction and Results sections to improve the overall flow of the manuscript. The Introduction has been made more direct and focused, clarifying the study's motivation and providing a clearer transition to the Results section.

In the Results section, we removed the subsection "Equivalence of Asocial Dynamics" and incorporated its key elements at the beginning of the section to better contextualise the subsequent analysis. While we retained the overall ordering of the results to preserve the logical progression of the findings, we streamlined the text by removing details not essential to understanding the main results.

For instance, in "Collective perception with a minimalistic robot swarm", we removed portions of text discussing chemical-like reaction network transitions, as this perspective is introduced more explicitly in the following section, "Multiscale modelling: from macroscopic population dynamics to robot swarm experiments." We also shortened the text and made the sentences more direct to facilitate comprehension. Similarly, in the section "Fast, cohesive, accurate, and scalable collective decisions," we revised the text to emphasize the key observations while reducing detailed discussions of the parameter spaces in which these trends hold, thereby improving readability.

Overall, these revisions make the core contributions clearer and easier to follow.

2 - The manuscript focuses solely on conditions with two options. However, typical decision-making problems in natural or robotic systems often involve more than two options. Would the authors expect similar results in such cases? Any discussion or insights regarding the generalization of the proposed models to scenarios with a larger number of options would strengthen the manuscript.

To do - We agree with this point, which was also raised by Reviewer 1. In response, we have extended the study by adding a new subsection in the Results section entitled "Collective decision-making with more than two options", along with two figures: Figure 7 and Supplementary Figure SF12. These additions are described in detail in our response to Comment 4 of Reviewer 1.

3 - I had difficulty interpreting Figure 8. I assume that CI and DS represent cross-inhibition and direct-switch, but this should be explicitly stated. I assume that cross-inhibition uses type 2 in Figure 8 (A), but this is never explained in the caption. In the third two rows in Figure 8 (A), the manuscript states that "Direct-switch shows higher accuracy in the bottom-left region," but the figure itself appears to indicate higher accuracy in the right region. Higher accuracy compared to what baseline? In addition, I did not understand how the results shown in panels (B) and (C) agree with those in panel (A). It would be helpful if the authors could indicate which parts of Figure 8 (A) correspond to (B) and (C).

Response - We thank the reviewer for these important comments. We have clarified in the caption of Figure 8 (in revised version Figure 5) the short forms (CI/DS) and the cross-inhibition type: Comparison of the direct-switch **(DS)** and cross-inhibition **(CI) type-2** mechanisms ....

We have also clarified the point about accuracy in the figure caption: Direct-switch shows higher accuracy than cross-inhibition in the bottom-left region when the decision problem is easy ($q > 0.92$); however, this region is also associated with relatively low cohesion.

We thank the reviewers for identifying the inconsistency between panels (B) and (C) of Figure 8 (original manuscript). There was indeed a mismatch in the value of η_a. We have corrected the figures accordingly and, for consistency, we now keep η fixed to 0.05 for both $\eta_a = 0.5$ and $\eta_a = 0.1$ examined in Figure 8B and C. To improve the readability of the manuscript, these panels have been moved to the Supplementary Material (Figure SF11).

4 - I cannot find the Github page. Maybe, the authors are planning to make it public after the manuscript is accepted.

To do - We thank the reviewer for noting that the GitHub repository was private. The repository has now been made public and is accessible via the link provided in the manuscript.

Reviewer 3

This manuscript investigates how minimalistic robot swarms make collective decisions under various forms of biased or asocial dynamics – stubborn agents (zealots), corrupted communication, and independent environmental discovery – by comparing the direct-switch mechanism (weighted voter) with the bio-inspired cross-inhibition mechanism. The authors present a multiscale modelling framework comprising a simplified mean-field ODE system, a more detailed robot-specific ODE formulation, master-equation formulations for probabilistic simulations using the Gillespie algorithm, and extensive Kilobot-based swarm simulations. A key conceptual contribution is the elegant mathematical unification of different bias sources through a single set of parameters (η, η_a, η_b), showing that zealots, MITM-style message corruption, and self-sourcing can all be treated as manifestations of the same underlying noisy update process. This theoretical abstraction is appealing and enables a systematic exploration of how bias interacts with decision dynamics. The results, which demonstrate that cross-inhibition achieves faster, more cohesive, and more accurate decisions across a wider range of biased conditions than direct-switch, are consistent across the different modelling layers and clearly visualised through bifurcation diagrams, stationary probability distribution comparisons, and simulation heatmaps.

1 - **However, while the multiscale methodology is solid and the internal consistency between the models and simulations is convincing, several issues limit the strength of the conclusions. First, many of the qualitative findings such as the susceptibility of direct-switch to deadlock under noise, the robustness of cross-inhibition, and the improvement in accuracy due to moderate noise are conceptually in line with existing literature in swarm robotics, distributed decision-making, and biological collective behaviour. The manuscript extends these insights rather than fundamentally revising them.**

Response - We agree that several of the qualitative trends we report are consistent with those identified in the existing literature. However, we believe that this study makes a substantial contribution to the field in several important aspects, including unifying previously fragmented knowledge within a single multi-scale framework, examining the role of antagonistic noise in collective decision-making, and extending the analysis to multi-option problems while incorporating real-robot validation.

First, we introduce a multi-scale modelling framework that explicitly links microscopic interaction rules, macroscopic population dynamics, and robotic implementations. This unified perspective enables a consistent analysis of the underlying decision-making mechanisms and reveals equivalences that, to the best of our knowledge, have not previously been integrated within a single study. In addition, this formulation simplifies the analysis of the mean-field models and enables a systematic exploration of the parameter space across a broad range of asocial dynamics, including biased forms that are particularly relevant in real systems.

Second, we extend the existing body of work by explicitly examining the role of antagonistic noise. Although this form of asocial dynamics is expected to affect real artificial systems, it has received limited attention in swarm robotics and collective decision-making research. By incorporating it into both the analytical and robotic models, we can characterize its impact on system performance.

Finally, the revised manuscript now includes an analysis of the $n > 2$ case, as well as real-robot demonstrations, further strengthening the conclusions. Multi-option decision scenarios remain relatively underexplored in the literature.

2 - **Second, the generality of the proposed equivalence between different forms of asocial dynamics is somewhat overstated. The algebraic mapping is valid at the mean-field macro level, but it suppresses behavioural asymmetries: zealots are persistent state-holders, MITM attacks act per-message, and self-sourcing is state-dependent and embodiment-dependent. These distinctions may matter significantly in practice, particularly for finite or spatially structured systems, and should be more explicitly acknowledged. Some model–simulation mismatches, noted by the authors themselves (e.g., differences between simple vs. robot-specific ODE curves in Fig. 4), suggest that the simpler models only provide qualitative predictions, which in turn tempers the claim of broad applicability across biological systems.**

Response - We agree that the proposed equivalence holds at the mean-field and macroscopic levels and therefore does not capture all behavioural asymmetries. Such discrepancies are expected when relating simplified analytical models to embodied robotic systems: the goal is not to reproduce every implementation-specific detail, but rather to capture the dominant mechanisms governing the collective dynamics. In this respect, the models provide qualitative predictions of the system-level behaviour in presence of asocial dynamics. Importantly,

both our robot simulations and the newly added real-robot demonstrations exhibit trends consistent with the macroscopic predictions, despite hardware-induced noise and implementation asymmetries.

We have revised the manuscript to explicitly acknowledge these micro-level differences and to moderate the claims regarding the generality of the equivalence. Specifically, we now state the following in the Discussion:

This mean-field equivalence is inherently macroscopic: it captures how asocial influences bias population-level dynamics, but may mask implementation-specific asymmetries that become significant in finite or spatially structured swarms. Accordingly, analogies between robotic and natural systems should be interpreted primarily in qualitative terms. Nevertheless, the unified formulation helps identify key ingredients—such as inhibitory interactions—that can reshape collective dynamics and improve robustness under biased conditions.

4 - The most significant limitation, however, lies in the practical feasibility of implementation and the lack of physical robot experiments, which strongly affects the generalisability of the results. Nearly all algorithmic assumptions – single-neighbour polling, reliable and isolated message reception, unambiguous detection of conflicting opinions, uniform environmental sampling, and fixed-duration behavioural phases – are idealised and difficult to satisfy with real minimal robots such as Kilobots. Real systems experience overlapping broadcasts, packet collisions, line-of-sight effects, motor noise, spatial clustering, and heterogeneous sensing, none of which are reflected in the modelling. Additionally, the probability of self-sourcing is treated as a controllable parameter, yet in real embodied systems it emerges from movement, occlusion, and sensing dynamics, making it neither uniform nor tunable. Even zealots, as modelled, represent an idealised failure mode: real malfunctioning robots fail multimodally, often altering mobility or communication range rather than merely freezing their opinion. Without hardware experiments validating at least the qualitative trends, it remains unclear whether the observed superiority of cross-inhibition under noisy conditions would persist in a physically embodied swarm.

Response - We have validated the qualitative trends by conducting real-robot demonstrations using a swarm of 50 kilobots. Specifically, we performed 24 experiments, comprising three trials for each combination of n_a= 0.3, 0.5 for q=0.66 and q=0.92. The results are reported as black markers in Figures 3 and 4 and show good qualitative agreement with both the mathematical model and the robot simulations. These results help address the reviewer's concern regarding idealized algorithmic assumptions and their applicability to real minimal robotic systems. In addition, our robot simulations explicitly incorporate several sources of stochasticity and hardware-related variability: behavioral phase durations are sampled rather than fixed, message dissemination times vary across robots, and interactions are probabilistic. Moreover, each simulation run uses a different (though statistically uniform) environmental configuration. Together, these features relax idealized assumptions and better capture the constraints of real robotic platforms. Importantly, the qualitative agreement between simulation and physical robot experiments observed in our study is consistent with previous Kilobot-based collective decision-making works (e.g., [Zakir et al., IROS 2024]; [Reina et al., Communications Physics 2023]), which also report close correspondence between ARGoS simulations and real-robot performance. We have added a brief description of the robot experiments in the Methods section (lines 497-507).

5 - The code is not available at https://github.com/rainazakir/asocialdynamics; am receiving a 404 error.

Response - We thank the reviewer for noting that the GitHub repository was private. The repository has now been made public and is accessible via the link provided in the manuscript.

6 - Despite these limitations, the manuscript is clear, well written, and technically rigorous, and the unified bias framework is a genuine conceptual contribution. The multiscale alignment between ODEs, stochastic simulations, and robot simulations is commendable. However, the authors should temper their broader claims about biological generality and real-world applicability, and more explicitly discuss the constraints imposed by the idealised assumptions embedded in their modelling framework. Addressing these limitations through physical robot validation and a deeper analysis of how embodied noise and spatial structure might shift the stability landscape would strengthen the paper and its suitability for Nature Communications.

Response - We agree and have moderated these claims in the Discussion. Specifically, we now state that the equivalence among asocial dynamics is macroscopic and may not hold at the micro-level in finite or spatially structured systems, and that analogies to biological systems should therefore be interpreted qualitatively. We also

added explicit discussion of idealised assumptions, including simplified communication, sensing, and self-sourcing mechanisms, and clarified that the real-robot experiments validate qualitative trends rather than exact quantitative thresholds.

We thank the three reviewers and the editor for their helpful comments, which have improved our work.

Best regards,
Raina Zakir, Timoteo Carletti, Marco Dorigo and Andreagiovanni Reina

Responses to Reviewers

Manuscript ID : NCOMMS-25-73624

Submitted to Nature Communications

Bio-inspired decision making in robot swarms under biases

Raina Zakir[1,⋆] Timoteo Carletti[2] Marco Dorigo[1] Andreagiovanni Reina[3,4,∗]

[1]IRIDIA, Université Libre de Bruxelles, Brussels, Belgium,

[2]Department of Mathematics and Namur Institute for Complex Systems, naXys, University of Namur, Namur, Belgium

[3]Centre for the Advanced Study of Collective Behaviour, Universität Konstanz, Konstanz, Germany,

[4]Department of Collective Behaviour, Max Planck Institute of Animal Behavior, Konstanz, Germany

⋆ corresponding author, raina.zakir@ulb.be, andreagiovanni.reina@uni-konstanz.de

Dear Editor,

Below, we provide a complete list of the reviewers' comments along with our responses. Before detailing the revisions made to the second version of the manuscript, we would like to thank the reviewers for their constructive and insightful comments. We have carefully addressed all the points raised. Where appropriate, excerpts from the manuscript are shown in blue to clearly distinguish them from our responses. In cases where we believe no modification was necessary, we provide a clear and reasoned justification for our decision.

Reviewer 2

1 - **The authors have adequately addressed my previous comments. However, I have one minor comment regarding the structure of the manuscript. The figures should be cited in numerical order according to their appearance in the text. Currently, Figure 2 is referenced before Figure 1. It may not be necessary to refer to Figure 2 in the Introduction section.**

Response - We agree with the reviewer that figures should be cited in numerical order, and it is not necessary to refer to Figure 2A-B in introduction as we already discuss it in Results line 101-102. Therefore, we have removed reference to Figure 2A-B on line 36, 37 and 40 in introduction.

2 - **I attempted to install argos3-kilobot, but encountered a CMake version error and was unable to complete the setup. Therefore, I could not fully review or execute the code. However, I noticed that the README.md should be revised to match the directory names in the GitHub repository. Additionally, providing a README.md file for each simulation directory would improve clarity and make it easier to understand how to run the code.**

Response - We have significantly revised the GitHub README to improve clarity, now organizing it into four clear sections:

* 'Running Robot Simulations': Provides installation details required to run robot simulations, including clearer guidelines for installing argos and the argos3-kilobot plugin, the required compiler versions, along with common errors that can be encountered during installation and their solutions. It also describes the folder structure hosting the robot controller code for both simulations and real-robot experiments.

* 'Running Gillespie Simulations': Lists all dependencies and instructions needed to run the Gillespie simulations, now including a detailed description of all input parameters.

* 'Mathematical Analysis': Provides the Mathematica and MATLAB files that can be downloaded and run to generate the ODE data used in the study. The section now also highlights the package required.

* 'Plotting Data': Updated to match the current directory structure on GitHub, specifying which scripts were used to generate each the plots in the study.

Reviewer 3

I thank the authors for their detailed response to the reviewers and for the substantial revisions made to the manuscript. In my view, the revised version is considerably stronger than the original submission. The authors have addressed the main concerns raised in my previous review in a serious and constructive manner. My earlier concerns were mainly around the level of novelty, the breadth of some of the claims, the reliance on idealised models and simulations, and the lack of physical robot validation. The revised manuscript now makes the contribution clearer. I still do not see the paper as proposing a fundamentally new decision-making algorithm, since direct-switch and cross-inhibition are well-established mechanisms. However, the value of the paper is now better framed as a unified and systematic analysis of how different forms of asocial dynamics and bias affect collective decision-making in robot swarms. This is a reasonable and useful contribution, particularly because the analysis connects mean-field models, stochastic simulations, robot simulations, multi-option decision-making, and physical robot demonstrations. The addition of real-robot experiments is an important improvement. These experiments are necessarily limited in scope, but they help address my concern that the original manuscript relied too heavily on idealised assumptions and simulation results. I think the authors are right to present these experiments as qualitative validation rather than exhaustive hardware validation. The agreement between the physical experiments, robot simulations, and models strengthens the paper. I am also satisfied with the authors' response regarding the equivalence between different forms of asocial dynamics. The revised manuscript clarifies that this equivalence applies primarily at the macroscopic and model levels and that implementation-specific differences may still matter in finite, spatially structured, or physically embodied swarms. This caveat is important and should remain visible in the final version. The extension to decision-making with more than two options also improves the manuscript. It does not completely remove the fact that much of the paper is still built around the binary case, but it does show that the main qualitative trends are not restricted to best-of-2 decisions. This is sufficient for the current paper. I have only a few remaining minor comments.

1 - **The authors should continue to be careful not to overstate the physical validation. The experiments support the qualitative trends, but they do not validate the full parameter space or all forms of asocial dynamics considered in the modeling framework.**

Response - We have further clarified this by adding the following statement in line 155-156: These experiments focused on assessing whether the qualitative trends identified in simulation also emerge in physical robots under representative conditions, rather than on exhaustively covering the full parameter space.

We have also modified line 279 to state:

Crucially, in the representative conditions tested, experiments with 50 physical robots reproduce the qualitative trends predicted by the models and simulations, supporting the persistence of these effects under embodied non-idealities such as errors in motion and communication.

2 - **Some of the broader claims about biological systems and general decentralised decision-making systems should remain carefully phrased. The manuscript is strongest when it speaks about robot swarms and model systems. The biological analogies are useful, but should not be presented as direct validation of the model.**

Response - We thank the reviewer for the insightful comment, our manuscript identify the biological analogy as useful without drawing conclusions on the biological mechanisms. In line 267-268, we indicated: Accordingly, analogies between robotic and natural systems should be interpreted primarily in qualitative terms.

3 - **The speed comparison remains somewhat dependent on how undecided runs are treated. I do not think this requires new experiments, but the manuscript would benefit from a short clarification that the interpretation of decision speed is tied to the quorum-based stopping criterion and to how non-decisions are handled.**

Response - We have now added a clarification in line 209-212: Note that because decision times are right-censored, only runs that reached a decision within time T are included (see metric definitions in the Methods section Speed-accuracy and speed-cohesion metrics). Low-cohesion configurations may therefore be slower than reported, especially for direct-switch.

4 - **I have not performed a full code review and did not install or run the code. I therefore cannot independently assess the reproducibility of the results from the provided repository. I did inspect the repository briefly. It appears to contain code for the Gillespie simulations, robot simulations, plotting,**

Mathematica analyses, and Kilobot-related experiments. The repository also includes a README with some installation and execution instructions, including dependencies and scripts for generating simulation data and figures. This is useful, but without running the code I cannot comment on whether the instructions are sufficient in practice, whether all dependencies are correctly specified, or whether the paper's figures can be reproduced end-to-end. My assessment of the manuscript is therefore based on the paper, supplementary material, and response to reviewers, rather than on an independent verification of the code

Response - We have updated the GitHub repository to enhance the reproducibility of the reported results. Details of these revisions are provided in our response to Reviewer 2 (point 2).

We thank the reviewers and the editor for their helpful comments, which have improved our work.

Best regards,
Raina Zakir, Timoteo Carletti, Marco Dorigo and Andreagiovanni Reina
