# Supplementary material for: Bio-inspired decision making in robot swarms under biases
Source: Nat Commun. 2026 Aug 14;17:9774. doi: 10.1038/s41467-026-76408-4 (PMC13572407; doi:10.1038/s41467-026-76408-4)
Supplement: Supplementary file 2 [file 41467_2026_76408_MOESM1_ESM.pdf]

Supplementary Materials of
Bio-inspired decision making in robot swarms under biases

Raina Zakir[1,*], Timoteo Carletti[2], Marco Dorigo[1], Andreagiovanni Reina [3,4,5,*]

[1]IRIDIA, Université Libre de Bruxelles, Brussels, Belgium
[2]Department of Mathematics and Namur Institute for Complex Systems, naXys, University of Namur, Namur, Belgium
[3]Centre for the Advanced Study of Collective Behaviour, Universität Konstanz, Konstanz, Germany
[4]Department of Computer and Information Science, Universität Konstanz, Konstanz, Germany
[5]Department of Collective Behaviour, Max Planck Institute of Animal Behavior, Konstanz, Germany
[*]raina.zakir@ulb.be, andreagiovanni.reina@uni-konstanz.de

Supplementary Text 1. Antagonistic bias in nature and artificial systems

Group-living animals, such as ants and bees, may encounter complex situations, such as the need to choose the best from multiple nest sites with differing qualities, prevent division when presented with identical potential sites, and reunify with the colony if a division may arise[1]. For instance, research has shown that ants can opt for the nest site with superior quality, even if it is located significantly farther away from their original nest than the site with lower quality (thus counteracting the antagonistic bias in easily discoverable near—but low-quality—site)[2,3]. Similarly, choosing an abundant foraging resource can increase the likelihood of attracting competitors, thereby exacerbating the antagonistic bias and incurring additional costs for defending the resources[4,5].

In artificial systems, previous studies have investigated the problem of synergistic and antagonistic environmental biases, which can lead robot swarms to select suboptimal options. In the context of synergistic bias,[6] studied a foraging scenario where the swarm's objective was to forage from the closest patch, showcasing a synergistic interplay, as the distance served as both option quality—since robots could measure it directly—and option cost, as closer resources were easier to discover. Other works, such as[7,8], have examined cue-based aggregation where the quality and size of each aggregation spot is proportional to light luminance, generating a synergistic bias because brighter spots (higher quality) also have larger areas (lower cost), making them easier for robots to discover.

Regarding antagonistic bias, Campo et al.[9] studied a scenario in which the robots were programmed with a collective decision-making algorithm inspired by cockroach aggregation behaviour, operating in an environment with two shelters of varying sizes. Shelter size determined quality and cost: small shelters could not accommodate the full swarm (higher cost), while larger shelters were more accessible (lower cost) but potentially of lower quality. In one of their experimental setups, the swarm's objective was to select the smallest shelter that could fit all robots, resulting in an antagonistic interplay between quality and cost. In Amé et al.[10], robots coated with pheromone were deployed in a cockroach shelter selection scenario, making them appear conspecific to the cockroaches. Unlike the cockroaches, the robots were programmed to prefer lighter shelters. As a result, the robots socially influenced the cockroaches to choose the lighter shelter, deviating them from their natural behaviour. In Reina et al.[11], Cai et al.[12], and Cody et al.[13], the authors investigated a binary foraging scenario in which a robot swarm used the cross-inhibition algorithm to collectively identify the foraging patch with the highest-quality resource. The environment included a home area and two foraging patches with varying resource qualities, which robots could measure using their sensors. The spatial arrangement of the two foraging patches introduced an antagonistic relationship between option quality and cost, where the highest-quality patch was located farthest from the home area, making it harder to discover and reach. In Reina et al.[11], consensus for the highest-quality option was achieved by slowing the decision-making process, allowing the formation of representative proportions of agents supporting either option that mitigated the effect of environmental bias, up to certain limits. Similarly, Cody et al.[13] used interaction delays as a mechanism to regulate how robots perceived the cost and quality of options. In De Masi et al.[14] and Antonic et al.[15], the swarm comprised zealots that systematically supported the inferior option. De Masi et al.[14] mitigated the impact of zealots by introducing informed agents who, unlike the uninformed agents, could estimate the quality more accurately from the environment. Antonic et al.[15] used heterogeneity in agent behaviour—different voting mechanisms—to regulate a cost-resiliency trade-off.

The presence of asocial dynamics can sway consensus dynamics towards inferior options in swarms; however, when introduced in moderate amounts, it can also serve as a control mechanism to achieve beneficial outcomes. For instance, the presence of a minority of zealots can prevent the swarm from becoming locked in outdated beliefs[16]. Similarly, a minority of informed robots can enable the flocking of the swarm towards a target direction, even when the target shifts[17]. A small number of informed robots can also modulate the distribution of robots across aggregation sites[18,19].

There are studies that, similar to our research, also consider the collective perception scenario where the quality of

two options is implicitly represented by the proportion of tiles in each of the two colours. However, in those studies, the environmental bias arises from how tiles of the same colour are spatially clustered[20], whereas in our case, tiles representing the two options—each with explicitly different qualities—are uniformly distributed in the environment. Additionally, some studies employed complex Bayesian hypothesis models to compute option qualities. Such models are not well suited to minimalistic swarms, as they require higher robot capabilities in terms of memory, computation, and communication[21].

Supplementary Text 2. Robot-specific ordinary differential equations

We extend the basic ODEs introduced in Section *Mean-field models of the basic collective decision-making algorithm* of the main text to incorporate the exploration and dissemination phases of the proposed decision-making algorithm based on the implementation in the robots.

Let a_d, a_e, b_d, b_e, and u be the proportions of robots in the states A_D, A_E, B_D, B_E, and U, respectively.

Direct-switch. The generic model characterising the evolution of the proportions of robots employing the algorithm based on the direct-switch mechanism can be described by the following four equations ruling the evolution of four states:

$$\begin{cases} \frac{\mathrm{d}a_d}{\mathrm{d}t} = \dfrac{a_e}{t_e} - \dfrac{a_d}{q_a t_d} \\ \frac{\mathrm{d}b_d}{\mathrm{d}t} = \dfrac{b_e}{t_e} - \dfrac{b_d}{q_b t_d} \\ \frac{\mathrm{d}a_e}{\mathrm{d}t} = -\dfrac{a_e}{t_e} + \left(\dfrac{a_d}{q_a t_d} + \dfrac{b_d}{q_b t_d}\right)\left(\eta\eta_a + (1-\eta)\dfrac{a_d}{a_d+b_d}\right) \end{cases} \tag{1}$$

where b_e= 1-a_d-b_d-a_e.

Cross-inhibition. The generic model characterising the evolution of the proportions of robots employing the algorithm based on the cross-inhibition type-1 mechanism, comprising five states described by four ODEs, is given by:

$$\begin{cases} \frac{\mathrm{d}a_d}{\mathrm{d}t} = \dfrac{a_e}{t_e} - \dfrac{a_d}{q_a t_d} \\ \frac{\mathrm{d}b_d}{\mathrm{d}t} = \dfrac{b_e}{t_e} - \dfrac{b_d}{q_b t_d} \\ \frac{\mathrm{d}a_e}{\mathrm{d}t} = -\dfrac{a_e}{t_e} + \left(\dfrac{a_d}{q_a t_d} + \dfrac{u}{t_u}\right)\left(\eta\eta_a + (1-\eta)\dfrac{a_d}{a_d+b_d}\right) \\ \frac{\mathrm{d}b_e}{\mathrm{d}t} = -\dfrac{b_e}{t_e} + \left(\dfrac{b_d}{q_b t_d} + \dfrac{u}{t_u}\right)\left(\eta\eta_b + (1-\eta)\dfrac{b_d}{a_d+b_d}\right) \end{cases} \tag{2}$$

where u = 1-a_d-b_d-a_e-b_e.

As discussed in Section *Equivalence of asocial dynamics* of the main text, the generic model can be instantiated for the three kinds of asocial dynamics: zealots, communication errors, or self-sourced opinions. For both direct-switch and cross-inhibition models, when we consider asocial dynamics in the form of zealots, we have $\eta = (z_a+z_b)/(a_d+b_d+z_a+z_b)$ as the probability to interact with zealots, $\eta_a = z_a/(z_a+z_b)$ as the probability to meet a zealot sharing option A, and $\eta_b = z_b/(z_a+z_b)$ as the probability to meet a zealot sharing option B. We prove the mathematical equivalence between the three kinds of asocial dynamics in Section 5.

In both direct-switch and cross-inhibition models, the proportions of robots disseminating an option, either robots in state A_D disseminating option A or B_D robots disseminating option B, increase at a rate t_e^{-1} due to robots transitioning from the exploration state to the dissemination state. These proportions also decrease at a rate $(q_a t_d)^{-1}$ for A_D (or $(q_b t_d)^{-1}$ for B_D) as robots leave the dissemination state after polling or after self-sourcing.

Next, consider the proportions a_e and b_e of robots exploring option A or B, respectively. The dynamics of agents in exploration state depend on the decision mechanism (either the direct-switch or cross-inhibition).

In the case of the direct-switch mechanism, the fraction of robots exploring option A as a_e (or for option B as b_e) decreases at a rate t_e^{-1} as agents move from the exploration to the dissemination state. The robots exploring option A as a_e (or option B as b_e) increase through several pathways: (i) when a robot disseminating for B (or A) adopts option A (or B) under social influence and transits to the exploration state A_E (or B_E) at a rate $(a_d/(a_d+b_d))(1-\eta)(q_b t_d)^{-1}$ (or $(b_d/(a_d+b_d))(1-\eta)(q_a t_d)^{-1}$); (ii) when a robot disseminating for option A (or B) does not change its opinion and enters the exploration state as A_E (or B_E) at a rate $(a_d/(a_d+b_d))(1-\eta)(q_a t_d)^{-1}$ (or $(b_d/(a_d+b_d))(1-\eta)(q_b t_d)^{-1}$); and (iii) when robots disseminating for option B or option A incur asocial dynamics at a rate $(\eta\eta_a)(q_b t_d)^{-1}$ or $(\eta\eta_a)(q_a t_d)^{-1}$, respectively.

In the case of the cross-inhibition mechanism, exploration proportions a_e and b_e also decrease at a rate t_e^{-1} when robots transit from the exploration to the dissemination state. One way the exploring robots a_e (or b_e) increase is through social or asocial recruitment of uncommitted robots U. Specifically, uncommitted robots adopt opinion A (or B) and enter the corresponding exploration states as A_E (or B_E) with a rate $(a_d/(a_d+b_d))(1-\eta)(t_u)^{-1}$ (or $(b_d/(a_d+b_d))(1-\eta)(t_u)^{-1}$) when

they incur social information, or with rates $(\eta\eta_a)(t_u)^{-1}$ (or $(\eta\eta_b)(t_u)^{-1}$) when they incur asocial information. Additionally, robots disseminating for option A (or B) may enter the exploration state as a_e (or b_e) for their respective option either through social dynamics—at a rate $(a_d/(a_d+b_d))(1-\eta)(q_a t_d)^{-1}$ (or $(b_d/(a_d+b_d))(1-\eta)(q_b t_d)^{-1}$)—or through asocial dynamics to update their opinion at a rate $(\eta\eta_a)(q_a t_d)^{-1}$ (or $(\eta\eta_b)(q_b t_d)^{-1}$).

The speed of the collective decision-making process described by the ODE models is determined by the magnitude and ratio of the control parameters q_a, q_b, t_d, t_e and, in the case of cross-inhibition, t_u.

Under the cross-inhibition mechanism, before adopting a new opinion, individuals must pass through the intermediate state U upon encountering environmental evidence that conflicts with their current opinion (we refer to this mechanism as cross-inhibition type-1). To enable a more comprehensive comparison of direct-switch and cross-inhibition, we also examine a second type of cross-inhibition mechanism. This mechanism, called cross-inhibition type-2, follows the self-sourcing implementation in the direct-switch mechanism, wherein a robot immediately adopts the environmental evidence it encounters, bypassing the uncommitted state. The basic ODE system for the cross-inhibition type-2 mechanism with generic asocial dynamics reads as

$$\begin{aligned} \tfrac{\mathrm{d}a}{\mathrm{d}t} &= a(1-\eta)(q_a u - q_b b) + \eta(b\eta_a - a\eta_b) \\ \tfrac{\mathrm{d}b}{\mathrm{d}t} &= b(1-\eta)(q_b u - q_a a) + \eta(a\eta_b - b\eta_a) \end{aligned} \tag{3}$$

This basic model can be extended in the robot-specific form as:

$$\begin{cases} \frac{\mathrm{d}a_d}{\mathrm{d}t} = \frac{a_e}{t_e} - \frac{a_d}{q_a t_d} \\ \frac{\mathrm{d}b_d}{\mathrm{d}t} = \frac{b_e}{t_e} - \frac{b_d}{q_b t_d} \\ \frac{\mathrm{d}a_e}{\mathrm{d}t} = -\frac{a_e}{t_e} + \left(\frac{a_d}{q_a t_d} + \frac{u}{t_u}\right)\left(\eta\eta_a + (1-\eta)\frac{a_d}{a_d+b_d}\right) + \eta\eta_a\frac{b_d}{q_b t_d} \\ \frac{\mathrm{d}b_e}{\mathrm{d}t} = -\frac{b_e}{t_e} + \left(\frac{b_d}{q_b t_d} + \frac{u}{t_u}\right)\left(\eta\eta_b + (1-\eta)\frac{b_d}{a_d+b_d}\right) + \eta\eta_b\frac{a_d}{q_a t_d} \end{cases} \tag{4}$$

Henceforth, we will refer to this second mechanism defined by Equations (4) as cross-inhibition type-2, and the first mechanism defined by Equations (2) as cross-inhibition type-1. In the direct-switch mechanism, as there is no uncommitted state U, the opinion update always follows the type-2 pattern.

Supplementary Text 3. Proof of the existence of a unique equilibrium

3.1 Direct-switch

Let us consider the basic ODE system for the direct-switch mechanism with generic asocial dynamics

$$\frac{da}{d\tau} = ab(1-\eta)(q_a - q_b) + \eta(b\eta_a - a\eta_b),$$

where $b = 1-a$. The aim of this section is to prove the existence of a unique physical equilibrium (a^*, b^*), namely with $0 < a^* < 1$ and $0 < b^* < 1$. The first step is to replace in the previous equations $b = 1-a$ to explicitly write the dependence on a:

$$\frac{da}{d\tau} = a(1-a)(1-\eta)(q_a - q_b) + \eta(\eta_a - a(\eta_a + \eta_b)).$$

The sought equilibrium, a^*, is a root of the right-hand side of the previous equation. Let us introduce two auxiliary functions

$$h_1(a) = a(1-a)(1-\eta)(q_a - q_b) \text{ and } h_2(a) = \eta(a(\eta_a + \eta_b) - \eta_a),$$

then it is clear that a^* is such that $h_1(a^*) = h_2(a^*)$. Let us assume $q_a > q_b$ (see panel A of Figure 1), then $y = h_1(a)$ is a parabola with downward concavity, vanishing at $a = 0$ and $a = 1$. On the other hand, $y = h_2(a)$ represents a straight line with value $-\eta\eta_a < 0$ at $a = 0$ and $\eta\eta_b > 0$ at $a = 1$. By a geometrical argument, it is clear that the straight line must intersect the parabola only once in the interval $(0,1)$, and this intersection, a^* (green star), is the unique equilibrium point.

A very similar argument can be used in the case $q_b > q_a$ (see panel B of Figure 1). We can thus conclude that the direct-switch model allows one and only one physical equilibrium for any value of the parameters.

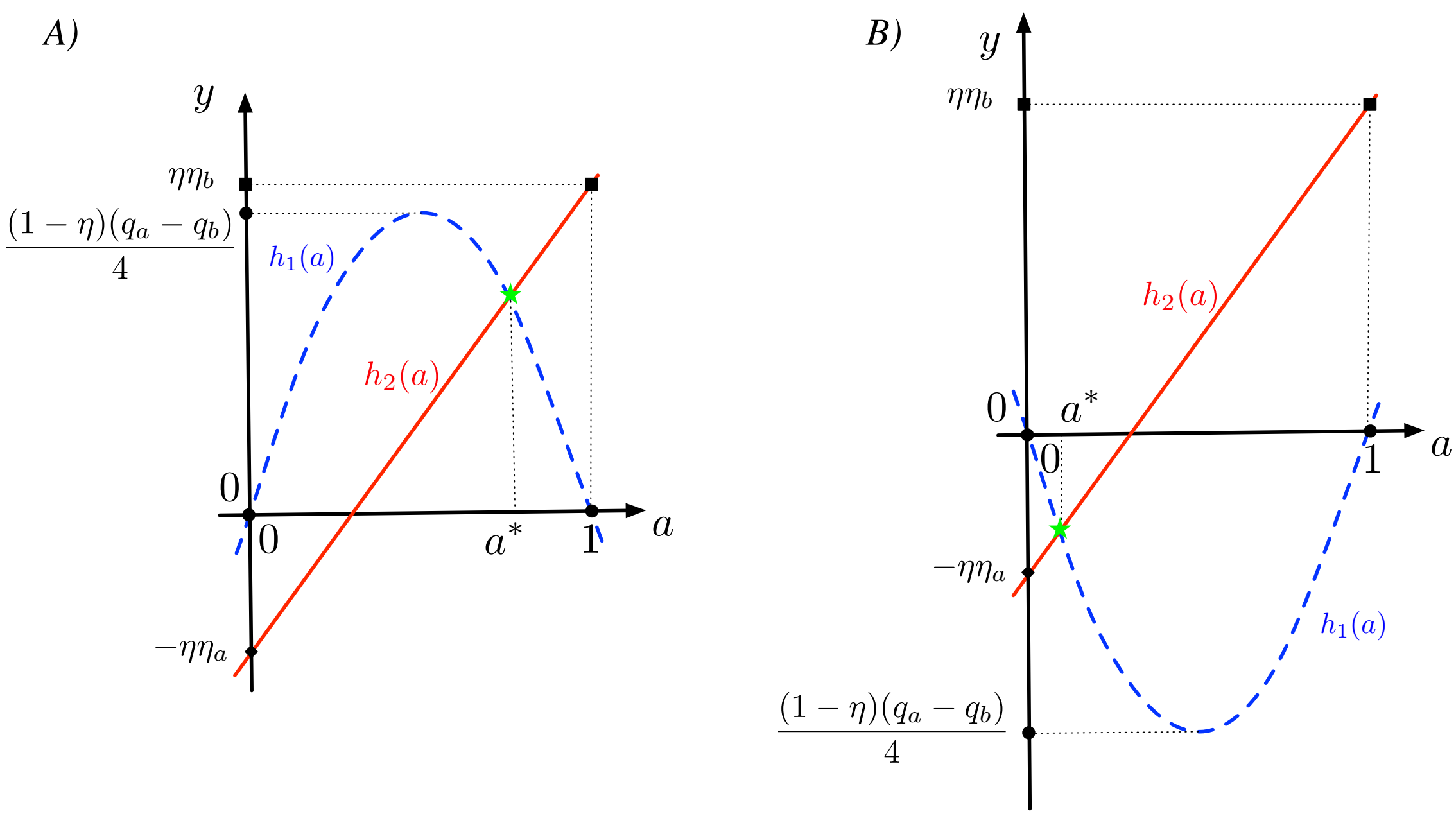

Supplementary Figure 1. Schematic representation of curves determining the possible equilibria of the direct-switch model in the plane (a, y).

3.2 Cross-inhibition

Let us now consider the ODE system for the cross-inhibition mechanism with generic asocial dynamics

$$\begin{aligned}\frac{da}{d\tau} &= a(1-\eta)(q_a u - q_b b) + \eta(u\eta_a - a\eta_b)\\ \frac{db}{d\tau} &= b(1-\eta)(q_b u - q_a a) + \eta(u\eta_b - b\eta_a)\end{aligned}$$

where $u = 1-a-b$. The aim of this section is to prove the existence of at least one physical equilibrium (a^*, b^*). The first step is to replace in the previous equations $u = 1-a-b$ to explicitly write the dependence on a and b

$$\begin{aligned}\frac{da}{d\tau} &= a(1-\eta)(q_a - q_a a - (q_a+q_b)b) + \eta(\eta_a - a(\eta_a+\eta_b) - \eta_a b)\\ \frac{db}{d\tau} &= b(1-\eta)(q_b - (q_a+q_b)a - q_b b) + \eta(\eta_b - a\eta_b - (\eta_a+\eta_b)b)\,.\end{aligned}$$

To have a qualitative view of the phase space, i.e., the plane (a,b), let us now consider the sets of points where each derivative vanishes, namely the 0-isocline functions:

$$\begin{aligned}f(a) &= \frac{(1-\eta)q_a a(1-a) + \eta\eta_a - \eta(\eta_a+\eta_b)a}{(1-\eta)(q_a+q_b)a + \eta\eta_a}\\ g(b) &= \frac{(1-\eta)q_b b(1-b) + \eta\eta_b - \eta(\eta_a+\eta_b)b}{(1-\eta)(q_a+q_b)b + \eta\eta_b}\,.\end{aligned}$$

One can thus realize that $\frac{da}{d\tau} = 0$ on the curve $b = f(a)$ and similarly $\frac{db}{d\tau} = 0$ for the points $a = g(b)$.

Let us now study the behaviour of the curve $b = f(a)$, similar conclusions could be drawn for the curve $a = g(b)$ (see panel A of Figure 2). First of all, by direct computation, we have

$$f(0) = 1 \text{ and } f(1) = \frac{-\eta\eta_b}{(1-\eta)(q_a+q_b)+\eta\eta_a} < 0\,,$$

where the inequality follows from the positivity of all involved quantities. This means that the curve $b = f(a)$ intersects the b-axis at 1 (black circle) and the vertical line $a = 1$ at some negative value (black square).

The roots of $f(a) = 0$ are given by the zeros of the numerator of $f(a)$. Because the latter is a second-degree polynomial, there can be 0 or 2 real roots. By a continuity argument, $f(a)$ is positive at $a = 0$ and negative at $a = 1$, there should be at least one root. By using a similar argument one can exclude the presence of a second root in $(0,1)$, because one should have $f(a)$ positive close to $a = 0$, then negative because of the first root and then positive again because of the second root, but we know that $f(a)$ should be negative close to $a = 1$ and thus a third root will be needed. This contradicts the number of zeros of the numerator of $f(a)$.

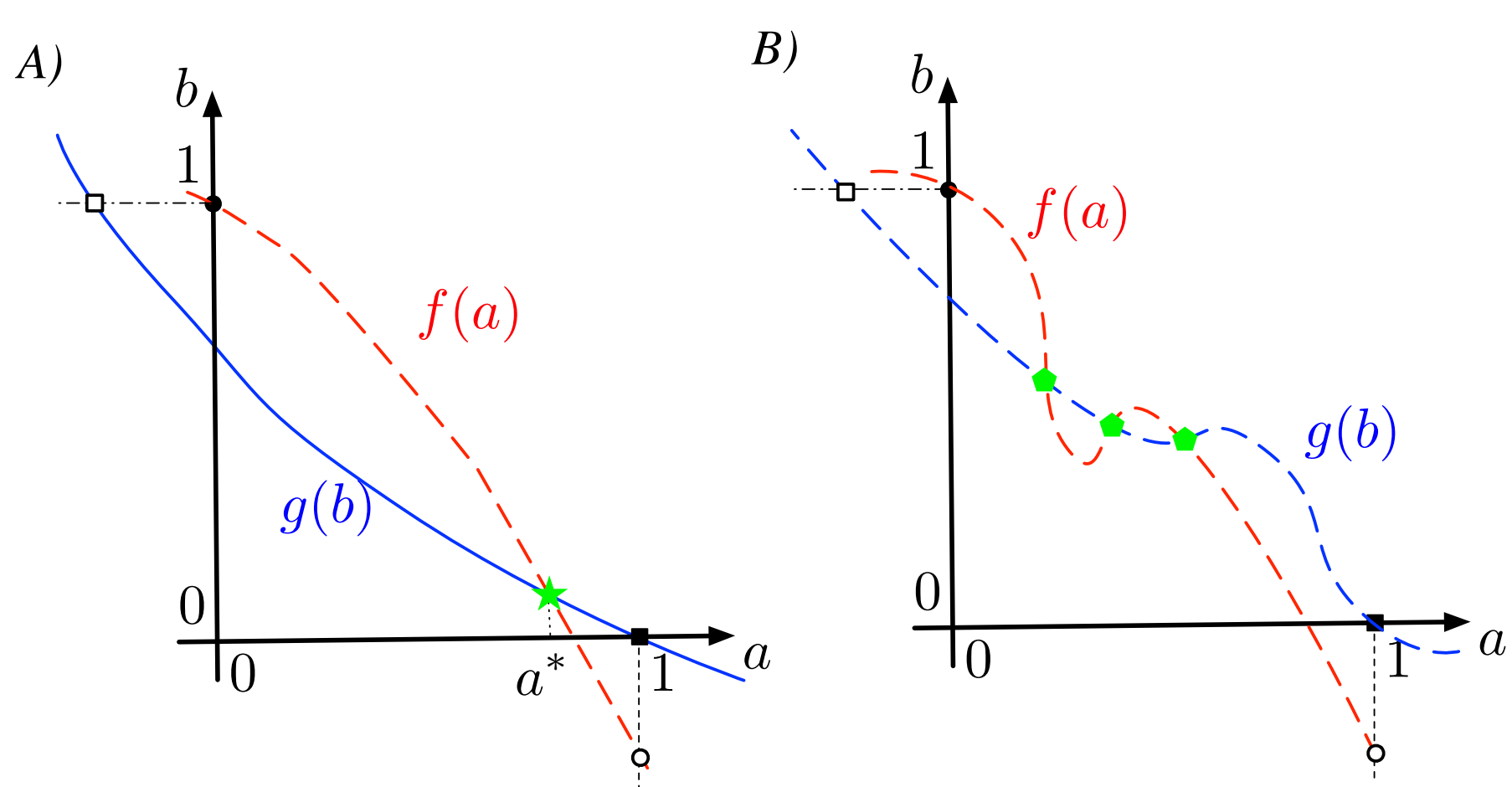

Supplementary Figure 2. Schematic representation of the phase space (a,b) and the 0-isoclines of the cross-inhibition model.

A similar analysis can be done for the curve $a = g(b)$ and we can thus conclude the existence of at least one physical equilibrium for the cross-switch model, a^* (green star). In panel B of Figure 2, we show another possible geometry for which three equilibria do exist (green pentagrams).

Supplementary Text 4. Critical bias for decision deadlocks

In the direct-switch mechanism, the decision deadlock occurs at a critical bias value η_a^* where both populations are in equal proportions:

$$\eta_a^* = \frac{-1+\eta+q+\eta q}{2\eta+2\eta q}\,, \tag{5}$$

where we assume $q_a = 1$. Equation (5) is derived from Equations (1), by setting the stable fixed points of the ODE system equal to each other, i.e., $A_D + A_E = B_E + B_D$. The deadlock point of Equation (5), marking the shift of majority between the two options, is shown as a black line in Figure 5 (last row) in the main text.

Supplementary Text 5. Equivalence of the asocial dynamics

The aim of this section is to show how the generic models can be instantiated to describe the three asocial dynamics. This section also emphasises the similarities among the three types of asocial dynamics, both in the direct-switch and cross-inhibition models.

5.1 Direct-switch

The generic model of direct-switch (Equations (1)) can be rewritten for zealots as follows:

$$\begin{cases} \frac{\mathrm{d}a_d}{\mathrm{d}t} = \frac{a_e}{t_e} - \frac{a_d}{q_a t_d} \\ \frac{\mathrm{d}b_d}{\mathrm{d}t} = \frac{b_e}{t_e} - \frac{b_d}{q_b t_d} \\ \frac{\mathrm{d}a_e}{\mathrm{d}t} = -\frac{a_e}{t_e} + \left(\frac{a_d}{q_a t_d} + \frac{b_d}{q_b t_d}\right) \frac{a_d + z_a}{a_d + b_d + z_a + z_b} \\ \frac{\mathrm{d}b_e}{\mathrm{d}t} = -\frac{b_e}{t_e} + \left(\frac{a_d}{q_a t_d} + \frac{b_d}{q_b t_d}\right) \frac{b_d + z_b}{a_d + b_d + z_a + z_b} \end{cases} \tag{6}$$

The generic model of direct-switch (Equations (1)) can be rewritten for man-in-the-middle (MITM) attacks as follows:

$$\begin{cases} \frac{\mathrm{d}a_d}{\mathrm{d}t} = \frac{a_e}{t_e} - \frac{a_d}{q_a t_d} \\ \frac{\mathrm{d}b_d}{\mathrm{d}t} = \frac{b_e}{t_e} - \frac{b_d}{q_b t_d} \\ \frac{\mathrm{d}a_e}{\mathrm{d}t} = -\frac{a_e}{t_e} + \left(\frac{a_d}{q_a t_d} + \frac{b_d}{q_b t_d}\right) \left(m m_a + (1-m)\frac{a_d}{a_d + b_d}\right) \\ \frac{\mathrm{d}b_e}{\mathrm{d}t} = -\frac{b_e}{t_e} + \left(\frac{a_d}{q_a t_d} + \frac{b_d}{q_b t_d}\right) \left(m m_b + (1-m)\frac{b_d}{a_d + b_d}\right) \end{cases} \tag{7}$$

The generic model of direct-switch (Equations (1)) can be rewritten for self-sourcing environmental information as follows:

$$\begin{cases} \frac{\mathrm{d}a_d}{\mathrm{d}t} = \frac{a_e}{t_e} - \frac{a_d}{q_a t_d} \\ \frac{\mathrm{d}b_d}{\mathrm{d}t} = \frac{b_e}{t_e} - \frac{b_d}{q_b t_d} \\ \frac{\mathrm{d}a_e}{\mathrm{d}t} = -\frac{a_e}{t_e} + \left(\frac{a_d}{q_a t_d} + \frac{b_d}{q_b t_d}\right) \left(\rho \rho_a + (1-\rho)\frac{a_d}{a_d + b_d}\right) \\ \frac{\mathrm{d}b_e}{\mathrm{d}t} = -\frac{b_e}{t_e} + \left(\frac{a_d}{q_a t_d} + \frac{b_d}{q_b t_d}\right) \left(\rho \rho_b + (1-\rho)\frac{b_d}{a_d + b_d}\right) \end{cases} \tag{8}$$

The models look identical once we make the following identifications among the coefficients:

$$\rho = m = \frac{z_a + z_b}{a_d + b_d + z_a + z_b}, \rho_a = m_a = \frac{z_a}{z_a + z_b}, \text{ and } \rho_b = m_b = \frac{z_b}{z_a + z_b}. \tag{9}$$

5.2 Cross-inhibition

The generic model of cross-inhibition type-1 (Equations (2)) can be rewritten for zealots as follows:

$$\begin{cases} \frac{\mathrm{d}a_e}{\mathrm{d}t} = -\frac{a_e}{t_e} + \left(\frac{a_d}{q_a t_d} + \frac{u}{t_u}\right) \frac{a_d + z_a}{a_d + b_d + z_a + z_b} \\ \frac{\mathrm{d}b_e}{\mathrm{d}t} = -\frac{b_e}{t_e} + \left(\frac{b_d}{q_b t_d} + \frac{u}{t_u}\right) \frac{b_d + z_b}{a_d + b_d + z_a + z_b} \\ \frac{\mathrm{d}a_d}{\mathrm{d}t} = \frac{a_e}{t_e} - \frac{a_d}{q_a t_d} \\ \frac{\mathrm{d}b_d}{\mathrm{d}t} = \frac{b_e}{t_e} - \frac{b_d}{q_b t_d} \\ \frac{\mathrm{d}u}{\mathrm{d}t} = -\frac{u}{t_u} + \frac{a_d}{q_a t_d} \frac{b_d + z_b}{a_d + b_d + z_a + z_b} + \frac{b_d}{q_b t_d} \frac{a_d + z_a}{a_d + b_d + z_a + z_b}. \end{cases} \tag{10}$$

The generic model of cross-inhibition type-1 (Equations (2)) can be rewritten for man-in-the-middle (MITM) attack as follows:

$$\begin{cases} \frac{\mathrm{d}a_e}{\mathrm{d}t} = -\frac{a_e}{t_e} + \left(\frac{a_d}{q_a t_d} + \frac{u}{t_u}\right) \left(m m_a + (1-m)\frac{a_d}{a_d + b_d}\right) \\ \frac{\mathrm{d}b_e}{\mathrm{d}t} = -\frac{b_e}{t_e} + \left(\frac{b_d}{q_b t_d} + \frac{u}{t_u}\right) \left(m m_b + (1-m)\frac{b_d}{a_d + b_d}\right) \\ \frac{\mathrm{d}a_d}{\mathrm{d}t} = \frac{a_e}{t_e} - \frac{a_d}{q_a t_d} \\ \frac{\mathrm{d}b_d}{\mathrm{d}t} = \frac{b_e}{t_e} - \frac{b_d}{q_b t_d} \\ \frac{\mathrm{d}u}{\mathrm{d}t} = -\frac{u}{t_u} + \frac{m m_a}{q_a t_d} a_d + \frac{m m_b}{q_b t_d} b_d + (1-m)\frac{a_d b_d}{a_d + b_d}\left(\frac{1}{q_a t_d} + \frac{1}{q_b t_d}\right). \end{cases} \tag{11}$$

The generic model of cross-inhibition type-1 (Equations (2)) can be rewritten for self-sourcing type-1 as follows:

$$\begin{cases} \frac{\mathrm{d}a_e}{\mathrm{d}t} = -\frac{a_e}{t_e} + \left(\frac{a_d}{q_a t_d} + \frac{u}{t_u}\right) \left(\rho \rho_a + (1-\rho)\frac{a_d}{a_d + b_d}\right) \\ \frac{\mathrm{d}b_e}{\mathrm{d}t} = -\frac{b_e}{t_e} + \left(\frac{b_d}{q_b t_d} + \frac{u}{t_u}\right) \left(\rho \rho_b + (1-\rho)\frac{b_d}{a_d + b_d}\right) \\ \frac{\mathrm{d}a_d}{\mathrm{d}t} = \frac{a_e}{t_e} - \frac{a_d}{q_a t_d} \\ \frac{\mathrm{d}b_d}{\mathrm{d}t} = \frac{b_e}{t_e} - \frac{b_d}{q_b t_d} \\ \frac{\mathrm{d}u}{\mathrm{d}t} = -\frac{u}{t_u} + \frac{\rho \rho_a}{q_a t_d} a_d + \frac{\rho \rho_b}{q_b t_d} b_d + (1-\rho)\frac{a_d b_d}{a_d + b_d}\left(\frac{1}{q_a t_d} + \frac{1}{q_b t_d}\right) \end{cases} \tag{12}$$

Let us first observe that the model involving self-sourcing type-2 (Equations (4)) is different from zealots or communication corruption because, in the former case, asocial dynamics make the robot immediately change opinion in favour of the other option, without passing through the uncommitted state. When interacting with a zealots or experiencing a communication errors, the robot cannot behave differently than from genuine social interactions because we assume the robot does not identify the asocial source of the information. It would otherwise reject it. Instead, when performing independent discovery, the robot is aware of the different information source and may or may not react in a different way (type-1 or type-2).

On the other hand, the models with zealots, communication errors, and self-sourcing type-1 can be mapped one onto the other by defining:

$$m = \rho = \frac{z_a + z_b}{a_d + b_d + z_a + z_b}, m_b = \rho_b = \frac{z_a}{z_a + z_b}, \text{ and } m_a = \rho_a = \frac{z_b}{z_a + z_b}$$

where m is probability of communication corruption, ρ is the probability to self-source. Indeed

$$\begin{aligned}\rho\rho_b(1-\rho)\frac{a_d}{a_d+b_d} &= \frac{z_a+z_b}{a_d+b_d+z_a+z_b}\frac{z_a}{z_a+z_b}+\left(1-\frac{z_a+z_b}{a_d+b_d+z_a+z_b}\right)\frac{a_d}{a_d+b_d} = \\ &= \frac{z_a}{a_d+b_d+z_a+z_b}+\frac{a_d+b_d}{a_d+b_d+z_a+z_b}\frac{a_d}{a_d+b_d} = \\ &= \frac{z_a}{a_d+b_d+z_a+z_b}+\frac{a_d}{a_d+b_d+z_a+z_b} = \frac{a_d+z_a}{a_d+b_d+z_a+z_b}\end{aligned}$$

Thus this proves that the left-hand side is equal to the right-hand side.

Supplementary Text 6. Equilibria for $n > 2$

We extend the basic ODE models presented in Section *Mean-field models of the basic collective decision-making algorithm* of the main text to the multi-alternative case with $n > 2$. The multi-alternative model for the direct-switch mechanism is given by

$$\dot{a}_i = a_i(1-\eta)\sum_{j\neq i} a_j(q_i - q_j) + \eta\sum_{j\neq i}\Big(a_j\eta_i - a_i\eta_j\Big), \qquad \forall i = 1,\ldots,n. \tag{13}$$

Instead the multi-alternative model for the cross-inhibition type-1 mechanism is given by

$$\dot{a}_i = a_i(1-\eta)\left(q_i u - \sum_{j\neq i} a_j q_j\right) + \eta\left(\eta_i u - a_i\sum_{j\neq i}\eta_j\right), \qquad \forall i = 1,\ldots,n. \tag{14}$$

In these models, a_i is the fraction of agents supporting the i-th option, η is the probability of receiving (generic) asocial information, while $1-\eta$ is the probability of receiving social information. In the cross-inhibition model, $u = 1 - \sum_i a_i$. In both models, η_i is the bias toward the i-th option when asocial information is received. In our best-of-n analysis, we set $\eta_i = 1/n$ for all $i = 1,\ldots,n$. The term q_i is the quality of the i-th option, and we assume $q_1 = 1$, $q_2 \in (0,1]$ and $q_j = pq_2$ where $j = 3,\ldots,n$. The parameter $p \in (0,1]$ regulates the relative qualities of the inferior options. We assume thus to have either two "main" options and several less "important" ones (when $p < 1$) or all inferior options with the same quality (when $p = 1$).

Supplementary Text 7. Chemical-like reaction network

Direct-switch. The direct-switch mechanism can be summarised using the following reaction scheme:

$$
\begin{aligned}
&A_D \to B_E \quad \text{rate: } \frac{1-\eta}{q_a t_d}\left(\frac{B_D}{A_D+B_D}\right) && B_D \to A_E \quad \text{rate: } \frac{1-\eta}{q_b t_d}\left(\frac{A_D}{A_D+B_D}\right)\\
&A_D \to A_E \quad \text{rate: } \frac{1-\eta}{q_a t_d}\left(\frac{A_D}{A_D+B_D}\right) && B_D \to B_E \quad \text{rate: } \frac{1-\eta}{q_b t_d}\left(\frac{B_D}{A_D+B_D}\right)\\
&A_E \to A_D \quad \text{rate: } \frac{1}{t_e} && B_E \to B_D \quad \text{rate: } \frac{1}{t_e}\\
&A_D \to B_E \quad \text{rate: } \frac{\eta\eta_b}{q_a t_d} && B_D \to A_E \quad \text{rate: } \frac{\eta\eta_a}{q_b t_d}\\
&A_D \to A_E \quad \text{rate: } \frac{\eta\eta_a}{q_a t_d} && B_D \to B_E \quad \text{rate: } \frac{\eta\eta_b}{q_b t_d}
\end{aligned}
\tag{15}
$$

Cross-inhibition. The cross-inhibition mechanism can be summarised using the following reaction scheme:

$$
\begin{aligned}
&A_D \to U \quad \text{rate: } \frac{1-\eta}{q_a t_d}\left(\frac{B_D}{A_D+B_D}\right) && B_D \to U \quad \text{rate: } \frac{1-\eta}{q_b t_d}\left(\frac{A_D}{A_D+B_D}\right)\\
&A_D \to A_E \quad \text{rate: } \frac{1-\eta}{q_a t_d}\left(\frac{A_D}{A_D+B_D}\right) && B_D \to B_E \quad \text{rate: } \frac{1-\eta}{q_b t_d}\left(\frac{B_D}{A_D+B_D}\right)\\
&U \to A_E \quad \text{rate: } \frac{1-\eta}{t_u}\left(\frac{A_D}{A_D+B_D}\right) && U \to B_E \quad \text{rate: } \frac{1-\eta}{t_u}\left(\frac{B_D}{A_D+B_D}\right)\\
&A_E \to A_D \quad \text{rate: } \frac{1}{t_e} && B_E \to B_D \quad \text{rate: } \frac{1}{t_e}\\
&A_D \to A_E \quad \text{rate: } \frac{\eta\eta_a}{q_a t_d} && B_D \to B_E \quad \text{rate: } \frac{\eta\eta_b}{q_b t_d}\\
&U \to A_E \quad \text{rate: } \frac{\eta\eta_a}{t_u} && U \to B_E \quad \text{rate: } \frac{\eta\eta_b}{t_u}\\
&A_D \to U \quad \text{rate: } \frac{\eta\eta_b}{q_a t_d} && B_D \to U \quad \text{rate: } \frac{\eta\eta_a}{q_b t_d} \quad \textbf{(self-sourcing type-1)}\\
&A_D \to B_E \quad \text{rate: } \frac{\eta\eta_b}{q_a t_d} && B_D \to A_E \quad \text{rate: } \frac{\eta\eta_a}{q_b t_d} \quad \textbf{(self-sourcing type-2)}
\end{aligned}
\tag{16}
$$

Supplementary Table 1. Model parameters and notation. The agent states A_D, B_D, A_E, B_E, and U also indicate the number of agents in that state when used as a population variable.

Symbol	Description
T	Simulation time steps
N	Swarm size
n	Number of options
q_i	Quality of option i
q_a	Quality of option A
q_b	Quality of option B
q	Quality ratio between q_b and q_a
a	Proportion of robots committed to option A
b	Proportion of robots committed to option B
u	Proportion of uncommitted robots
A_D	Agent state: disseminating and committed to opinion A
B_D	Agent state: disseminating and committed to opinion B
A_E	Agent state: exploring and committed to opinion A
B_E	Agent state: exploring and committed to opinion B
U	Agent state: uncommitted agent
t_e	Mean duration of exploration state
t_d	Mean duration of dissemination state
t_u	Mean duration of uncommitted state
η	Probability of opinion switching under asocial dynamics
η_i	Bias of asocial information toward option i
η_a^*	Critical bias for decision deadlocks
p	Relative quality of the inferior options
z	Number of zealots in the swarm
z_i	Proportion of zealots favouring option i
ρ	Probability of self-sourcing environmental information
ρ_i	Bias of self-sourcing toward option i
m	Probability of MITM attack
m_i	Bias of MITM attacks toward option i
T_c	Number of Kilogrid cells encountered by a robot
C_i	Number of cells matching option i encountered by a robot
σ	Sampling noise (standard deviation of quality estimates)
Q	Quorum threshold used in accuracy computation

Supplementary Algorithm 1. Robot Behaviour for Direct-Switch and Cross-Inhibition (type-2)

Parameters

η ▷ probability to self-source
η_a, η_b ▷ asocial bias — proportion of tiles
q_a, q_b ▷ nominal qualities
σ ▷ quality sampling noise
t_e, t_d, t_u ▷ mean phase durations
experimentType $\in \{$SYNERGETIC, ANTAGONISTIC$\}$
update_mode $\in \{$DIRECTSWITCH, CROSSINHIBITION$\}$

Initialization

opinion $\leftarrow$ **if** robot_id mod $2 = 0$ **then** A **else** B ▷ Even robot id $\Rightarrow$ option A, odd $\Rightarrow$ option B
state $\leftarrow$ EXPLORATION
$T_e \leftarrow$ sample Exponential(t_e)
if *experimentType* == SYNERGETIC **then**

```
    T_c ← 0, C_i ← 0, visited ← ∅                                  ▷ Initialize Kilogrid cells counters
else
    q_i ← 0
end if

Main Loop
for t = 0 to T do
    wall_flag ← readKilogridWallFlag()
    if wall_flag == HIGH then
        executeObstacleAvoidance()
    else
        executeRandomWalk()
    end if

    switch state do
        case EXPLORATION:
            if T_e > 0 then
                (cell_id, cell_colour) ← readKilogridCellIdColour()
                if experimentType == SYNERGETIC then
                    if cell_id ∉ visited then                       ▷ If the robot has not visited this cell yet
                        visited ← visited ∪ {cell_id}
                        T_c ← T_c + 1                               ▷ Count the number of visited cells
                        if cell_colour == opinion then
                            C_i ← C_i + 1      ▷ Count the number of cells with colour corresponding to the robot's opinion
                        end if
                    end if
                else                                                ▷ Antagonistic: sample quality on matching tile
                    if cell_colour == opinion then
                        q_i ← readKilogridCellQuality(σ)   ▷ Get option quality from Kilogrid with sampling noise σ
                    end if
                end if
                T_e ← T_e − 1
            else
                if experimentType == SYNERGETIC then                ▷ Compute quality from cell counts
                    q_i ← C_i / T_c
                end if
                state ← DISSEMINATION                               ▷ Change state
                if opinion == UNCOMMITTED then
                    T_d ← sample Exponential(t_u / 2)               ▷ Set uncommitted time
                else
                    T_d ← sample Exponential(q_i · t_d)             ▷ Set a quality dependent dissemination time
                end if
                opinion_neighbour ← ∅                               ▷ Reset variable
            end if

        case DISSEMINATION:
            if T_d > 0 then
                if opinion ≠ UNCOMMITTED then
                    broadcast(opinion)
                end if
                opinion_neighbour ← readIncomingMessage()
                T_d ← T_d − 1
            else
                u ← Uniform(0, 1)                                   ▷ Random number in the [0,1] range
```

```
                state ← { SelfSource   if u < η
                        { Polling      otherwise
            end if

        case Polling:
            if opinion_neighbour ≠ ∅ then
                if update_mode == DirectSwitch then
                    opinion ← opinion_neighbour                          ▷ Direct switch of opinions
                else if update_mode == CrossInhibition then
                    if opinion_neighbour ≠ opinion then
                        opinion ← Uncommitted                            ▷ Cross-inhibition
                    else
                        opinion ← opinion_neighbour                      ▷ Recruitment
                    end if
                end if
            end if
            state ← Exploration                                          ▷ Change state
            if opinion == Uncommitted then
                T_e ← sample Exponential(t_u/2)                          ▷ Set uncommitted time
            else
                T_e ← sample Exponential(t_e)                            ▷ Set exploration time
                if experimentType == Synergetic then
                    T_c ← 0, C_i ← 0, visited ← ∅                        ▷ Initialize Kilogrid cells counters
                else
                    q_i ← 0
                end if
            end if

        case SelfSource:
            opinion ← readKilogridCellColour()
            state ← Exploration                                          ▷ Change state
            T_e ← sample Exponential(t_e)                                ▷ Set exploration time
            if experimentType == Synergetic then
                T_c ← 0, C_i ← 0, visited ← ∅                            ▷ Initialize Kilogrid cells counters
            else
                q_i ← 0
            end if
    end switch
    t = t + 1
end for
```

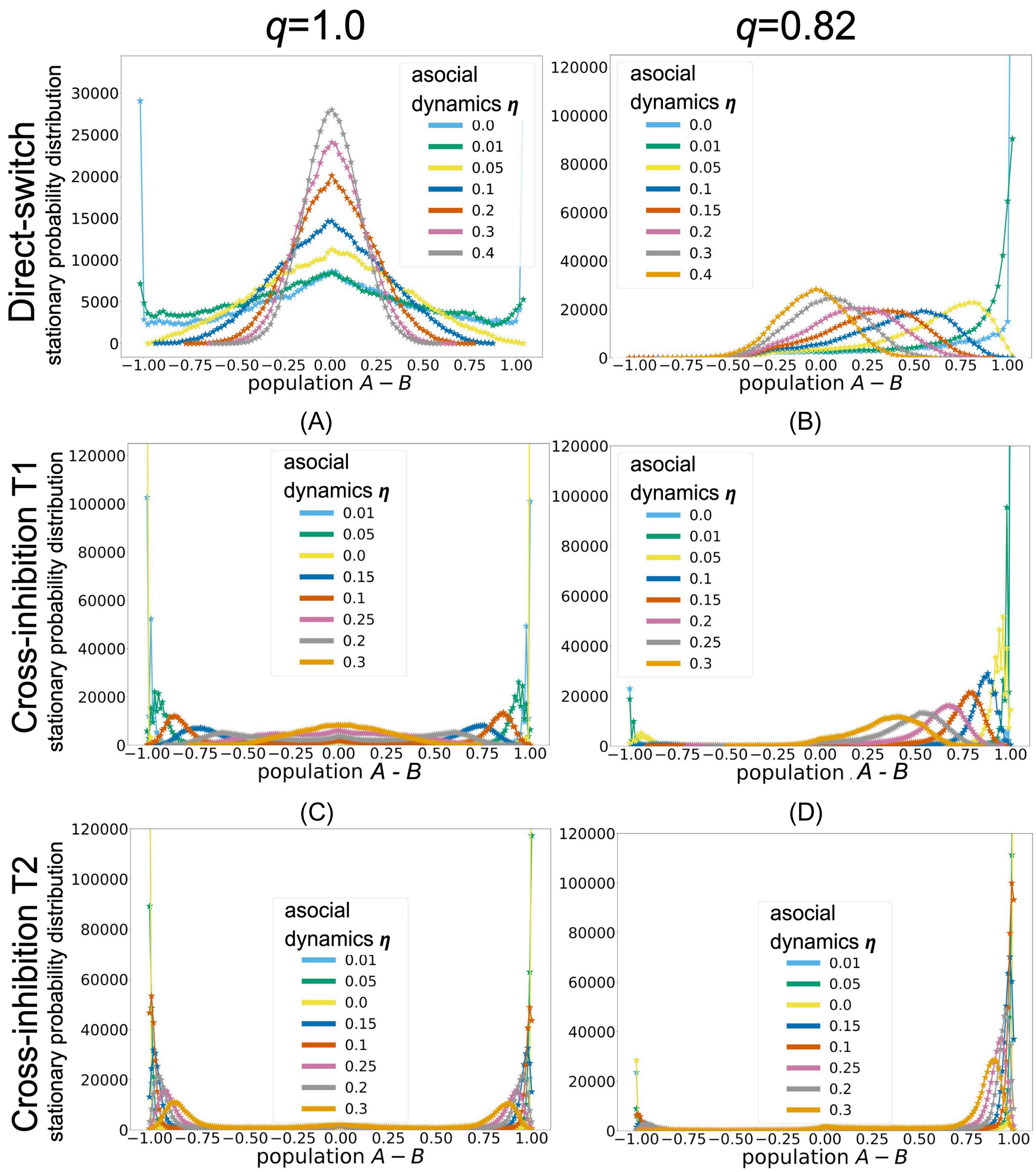

Supplementary Figure 3. Stationary probability distributions (SPDs) for several η, computed using Gillespie's stochastic simulation algorithm, are shown for same quality $q = 1$ (left column) and medium quality difference $q = 0.82$ (right column) for the direct-switch (top row) and cross-inhibition (bottom two rows). The plots show how the difference of robots supporting options A and B (x-axis) changes for various η. Each of the 250 Gillespie simulation runs per η begins with a random initial condition and records the time spent in each state during 200 000 time steps. The time spent in each state is normalised by the total simulation length to approximate the SPD. The obtained results are consistent with the numerical analysis of the histograms that are illustrated in the main text Figure 3 for both opinion update mechanisms. Direct-switch cannot break the symmetry for any level of noise ($\eta > 0$) when qualities are symmetric (A) and $\eta > 0.1$ when qualities are moderately similar (C). Instead, the cross-inhibition mechanism can break the symmetry for both symmetric (B) and asymmetrical qualities (D) unless the social noise is high ($\eta > 0.2$).

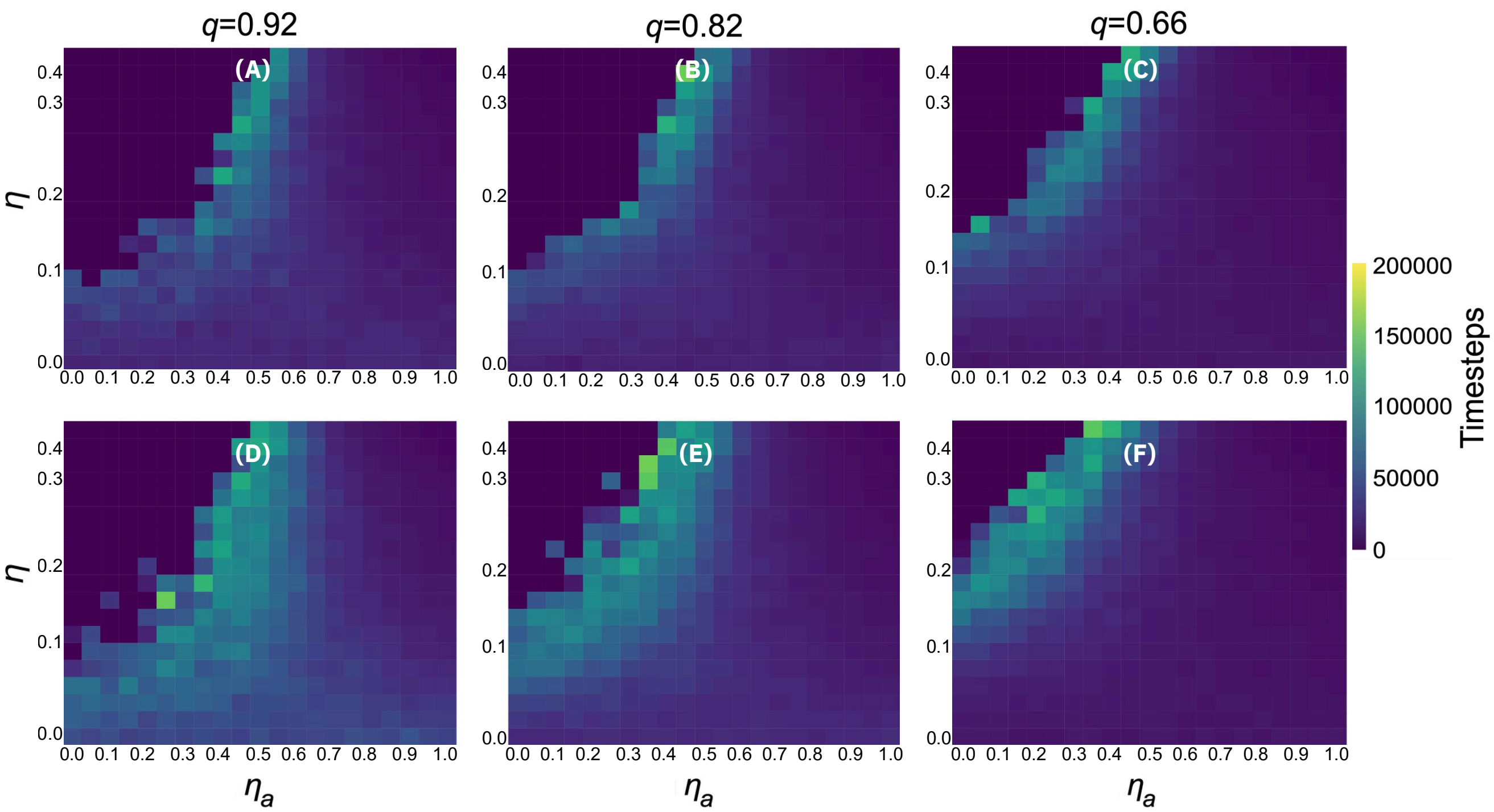

Supplementary Figure 4. Speed of reaching an accurate decision in the cross-inhibition type-2 (first row) and in the direct-switch (second row) for increasing self-sourcing noise η (y-axis) and increasing self-sourcing bias towards option A, η_a (x-axis), where $\eta_a = 0.5$ denotes the unbiased case, $\eta_a > 0.5$ denotes the synergistic case biased towards the best option A, and $\eta_a < 0.5$ denotes the antagonistic case biased towards the inferior option B. The heatmaps are generated using the Gillespie algorithm for quality differences of $q = 0.92$, $q = 0.82$, and $q = 0.66$. The results are based on a swarm of $N = 100$ agents with an initial condition of [A_D, B_D, A_E, B_E]=[14,14,36,36] and for different quality differences.

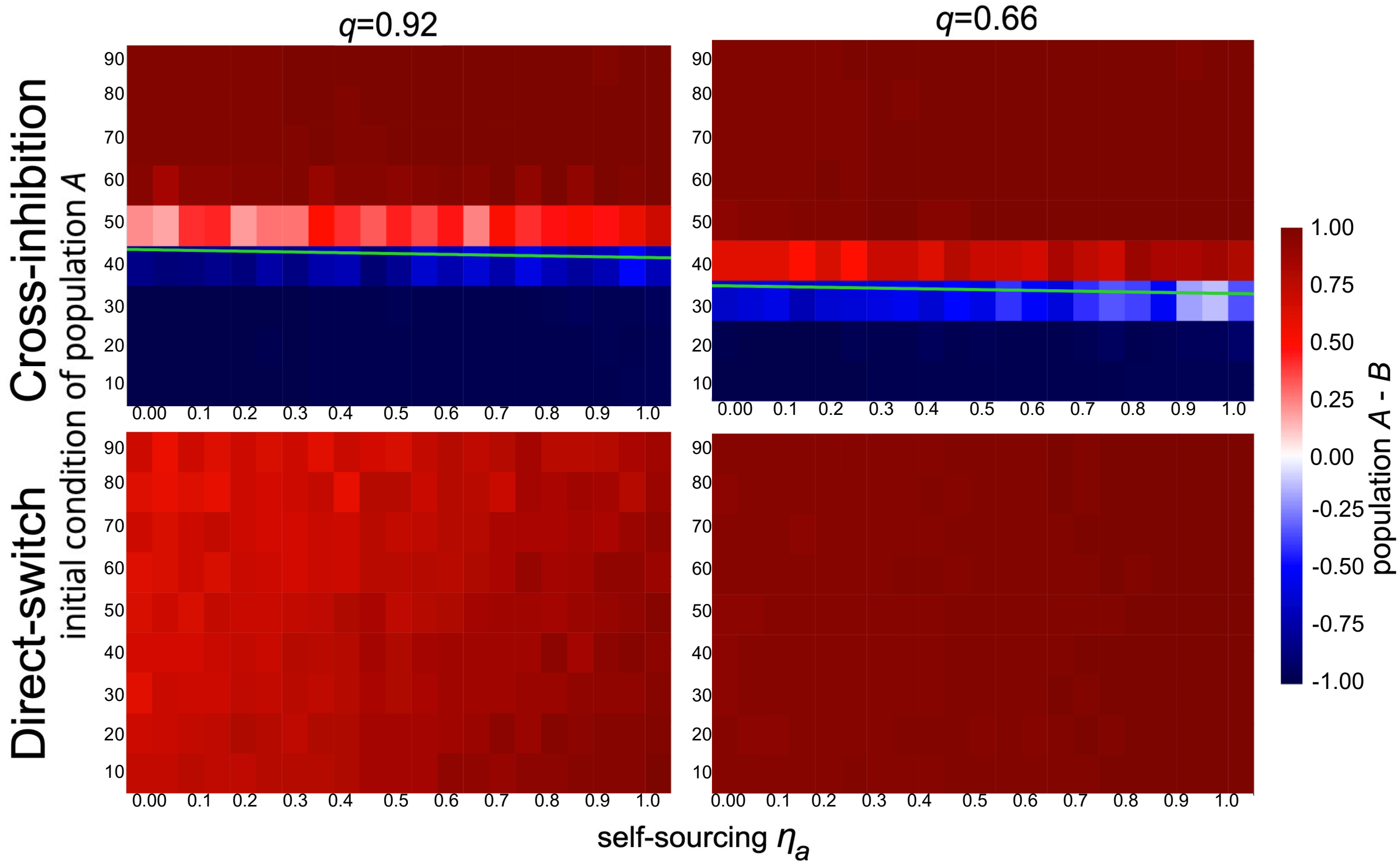

Supplementary Figure 5. Convergence to either option A or B across various initial condition of population A (y-axis), for cross-inhibition (type-2) and direct-switch, performed using 100 Gillespie simulations with $N = 100$ agents as a function of the self-sourcing bias for option A (η_a) (x-axis), for option quality $q = 0.92$ and $q = 0.66$ when $\eta = 0.01$. The green lines in the plots show the border that divides initial conditions that lead the system to consensus on option A (red area) from those with consensus on option B (blue area) as a function of η computed from the ODE model (Equations (2)).

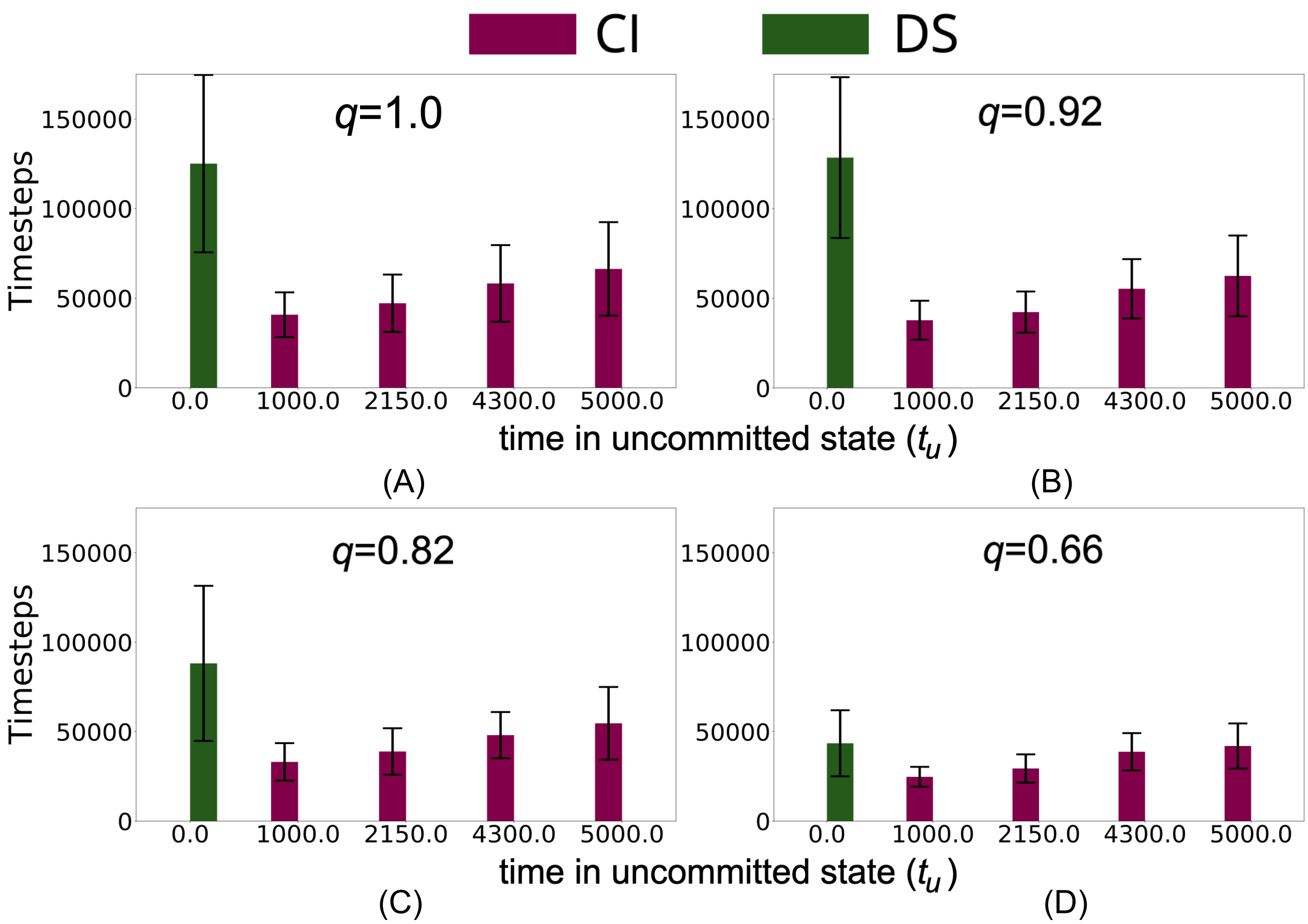

Supplementary Figure 6. Comparison of convergence speed to the higher-quality opinion A of the direct-switch (DS) and cross-inhibition type-2 (CI) mechanisms using three different uncommitted state times t_u (values on the x-axis). We report the results from 250 Gillespie simulations with $N = 100$ agents for four different q ratios, $\eta = 0.05$, and $\eta_a = 0.5$. These results show how the collective decision-making speed is influenced by the mean time t_u that robots spend in the uncommitted state. Figure 5 in the main text shows that cross-inhibition makes faster decisions, however this can be attributed to a relatively short time in the uncommitted state; in our experiment we use $t_u < t_d + t_e$. Therefore, we conduct additional experiments with $t_u = (t_d + t_e)/2 = 2150$, $t_u = t_d + t_e = 4300$, and $t_u > t_d + t_e = 5000$. The error bars represent the standard deviation in the convergence speed. The plots show that cross-inhibition's decision time increases moderately with t_u: a five-fold increase of t_u, from 1000 to 5000, resulted in less than doubling the decision time. In all cases, cross-inhibition remains faster in making decisions than direct-switch.

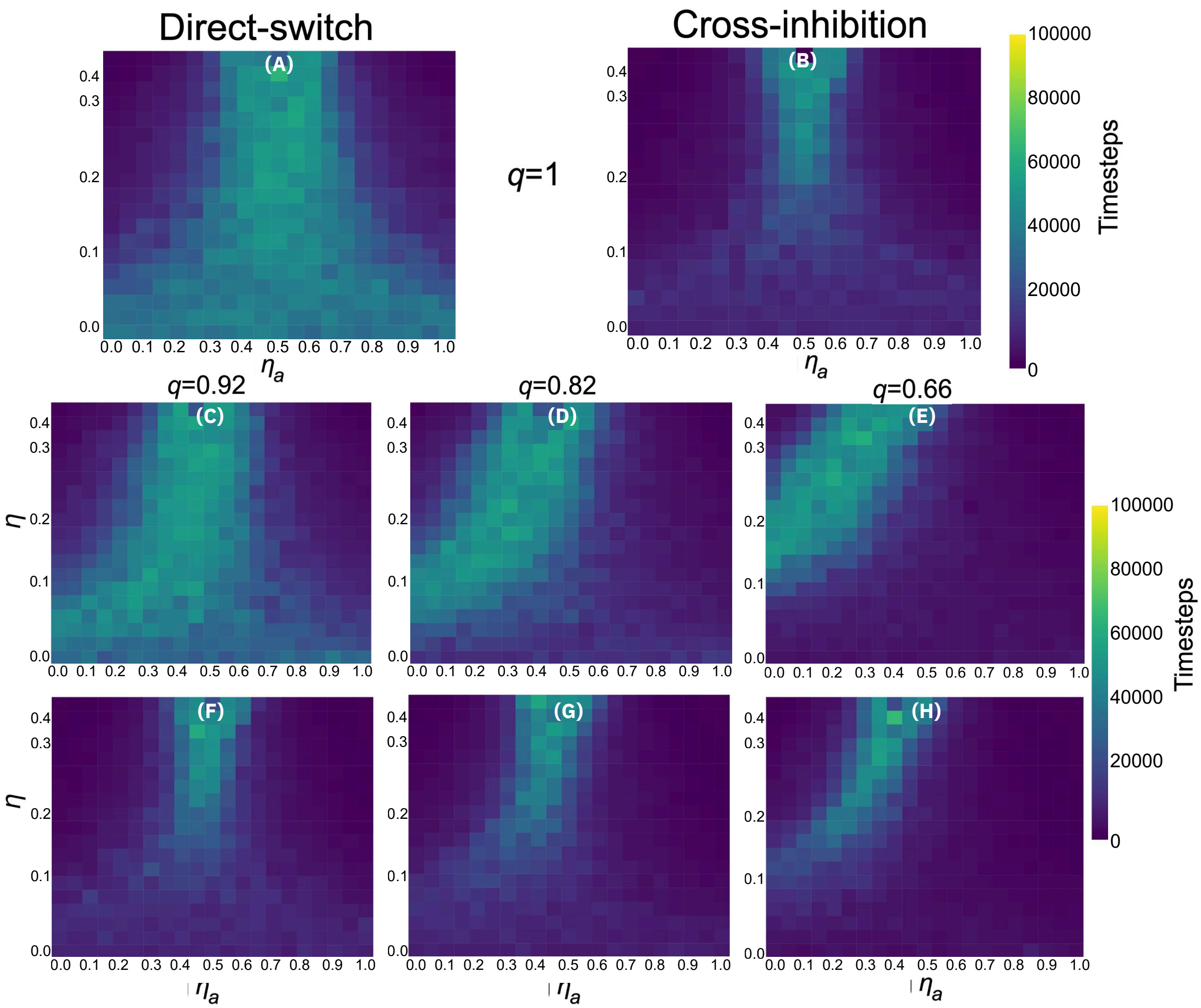

Supplementary Figure 7. Standard deviation of the decision speed for direct-switch (A,C,D,E) and for cross-inhibition type-2 (B,F,G,H) for increasing self-sourcing noise η (y-axis) and increasing self-sourcing bias towards option A, η_a (x-axis), where $\eta_a = 0.5$ denotes the unbiased case, $\eta_a > 0.5$ denotes the synergistic case biased towards the best option A, and $\eta_a < 0.5$ denotes the antagonistic case biased towards the inferior option B. The heatmaps were generated using the Gillespie algorithm for quality differences of $q = 0.92$, $q = 0.82$, and $q = 0.66$. The results are based on a swarm of N=100 agents with an initial condition of [A_D, B_D, A_E, B_E]=[14,14,36,36] and for various quality differences.

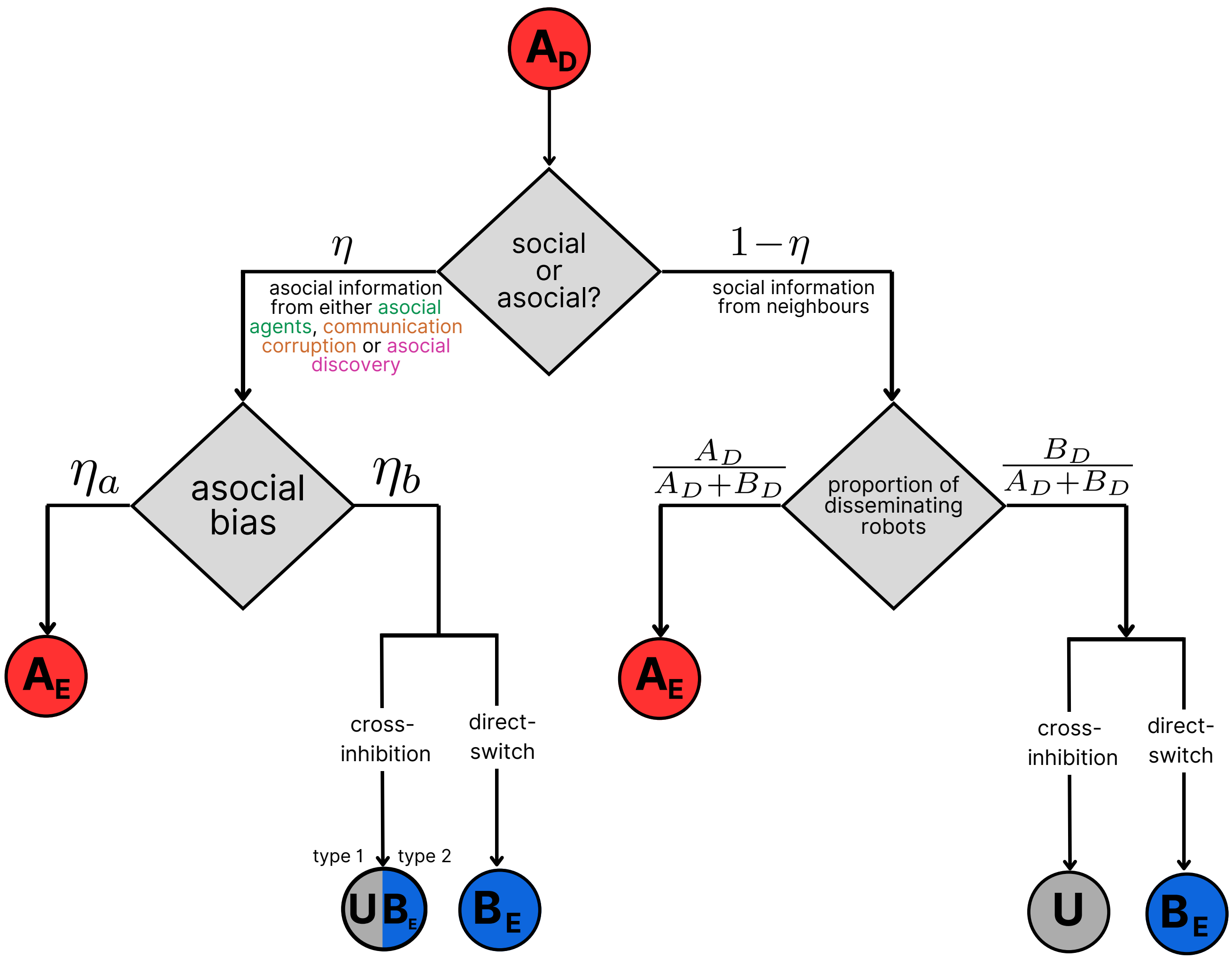

Supplementary Figure 8. Schematic representation of how a robot supporting option A updates its opinion at the end of the dissemination state. The new opinion depends on the information that the robot uses to update it. The robot either polls 'representative' social information with probability $1-\eta$ or is influenced by asocial dynamics with probability η. When the robot pools representative social information (right branch), the probability of reading a message supporting A or B is proportional to the proportion of disseminators for A (in state A_D) and B (in state B_D), respectively. Unlike Figure 1 in the main text, the transition rates here do not depend explicitly on q_a and q_b. Instead, quality dependence is captured implicitly through the composition of the disseminating subpopulations: the numbers of robots in A_D and B_D form a quality-weighted fraction of those committed to A and B, because dissemination time is proportional to the robot's estimated option quality. When influenced by asocial dynamics (left branch), such as self-sourcing, zealots, or communication corruption, the information can be biased towards A or B, with weights η_a and η_b, respectively. If the received information (whether social or asocial) is in support of A, the robot retains its opinion regardless of the opinion-update model. If the information supports B, the transition depends on the model: in the cross-inhibition model type-1, the robot becomes uncommitted (state U), while in the direct-switch model and cross-inhibition model type-2, it switches to exploring option B (state B_E).

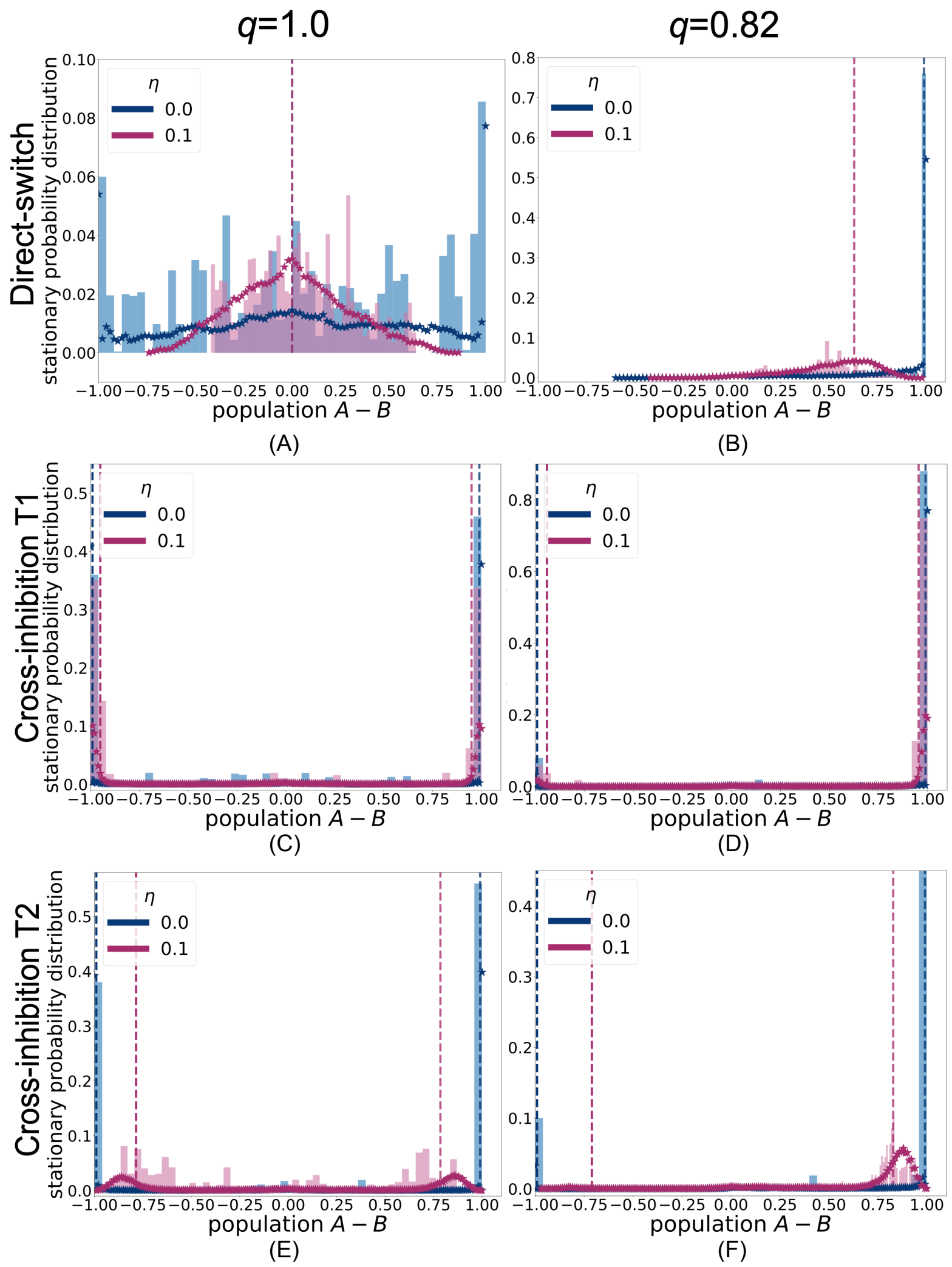

Supplementary Figure 9. Stationary probability distributions (SPDs) of $N = 100$ robots for $\eta = 0$ (no asocial dynamics) and $\eta = 0.1$ (moderate asocial dynamics), computed using Gillespie's stochastic simulation algorithm (star markers) and robot simulations (histograms). Stable equilibria (from ODE systems of Equations (1) and (2)) are overlaid as dashed vertical lines. The final distribution of robots (at stability) supporting A and B is consistent across the three analysis methods for two different η values. Each of the 50 Gillespie simulation runs per condition begins with a random initial condition and records the time spent in each state during 200 000 timesteps. The time spent in each state is normalised by the total simulation length to approximate the SPD. The histograms denoting the SPD from robot simulations are computed as the difference between the proportions A and B of robots supporting options A and B, respectively, for each of the last 1 000 timesteps of each of the 50 runs ($(A-B)/N$ on the x-axis). The direct-switch mechanism cannot break the symmetry for low and moderate noise when qualities are symmetric (A), but it never selects the inferior option (B) when qualities are asymmetric. Instead, the cross-inhibition mechanism can always break the symmetry (C,E) but occasionally selects the inferior option (D,F). The probability of choosing the inferior option diminishes in the presence of asocial dynamics ($\eta = 0$ vs $\eta = 0.1$ in D,F).

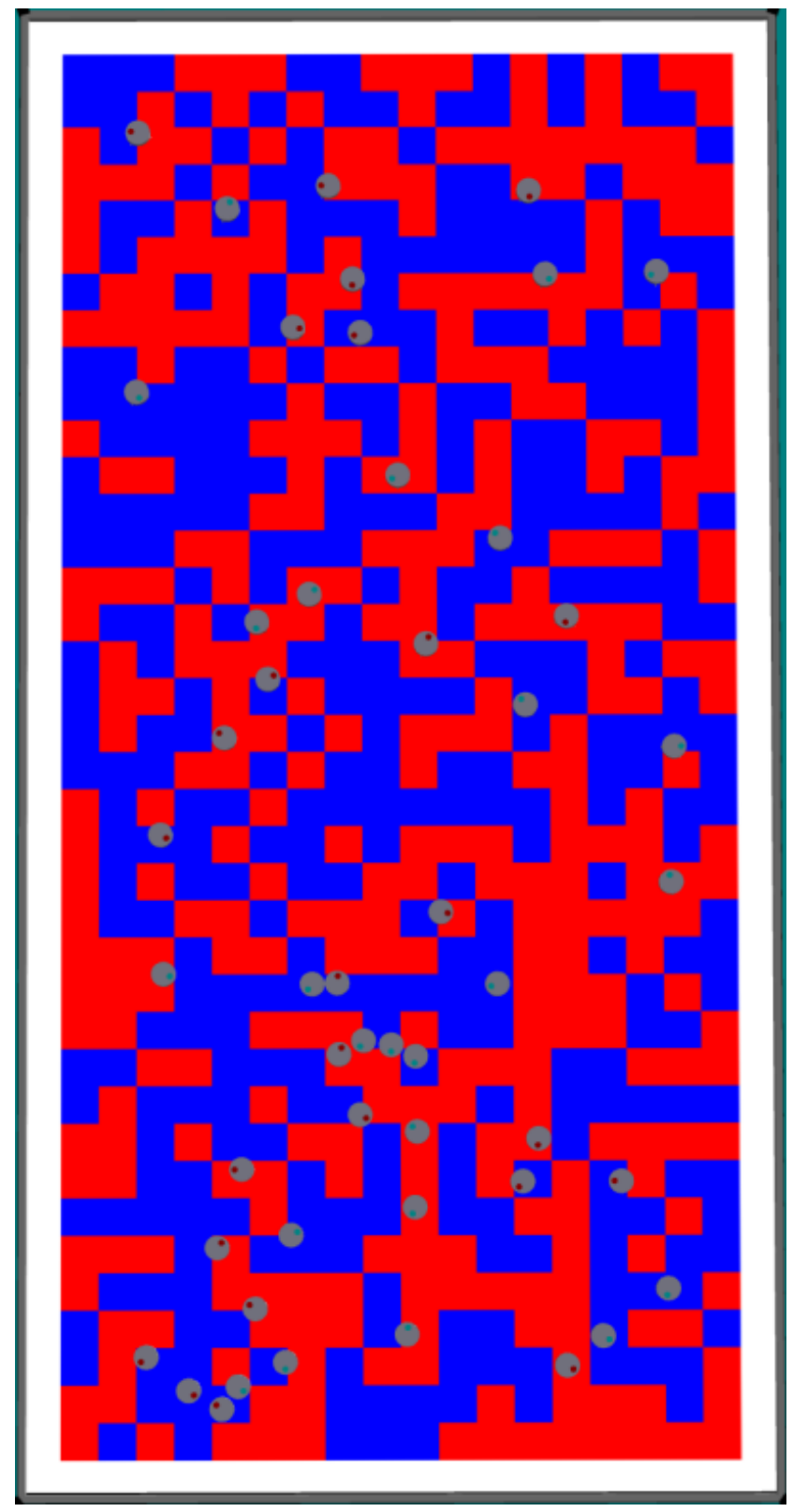

Supplementary Figure 10. Top-view snapshot of an experiment with 50 simulated Kilobots (grey circles) in the Kilogrid arena comprising red and blue tiles.

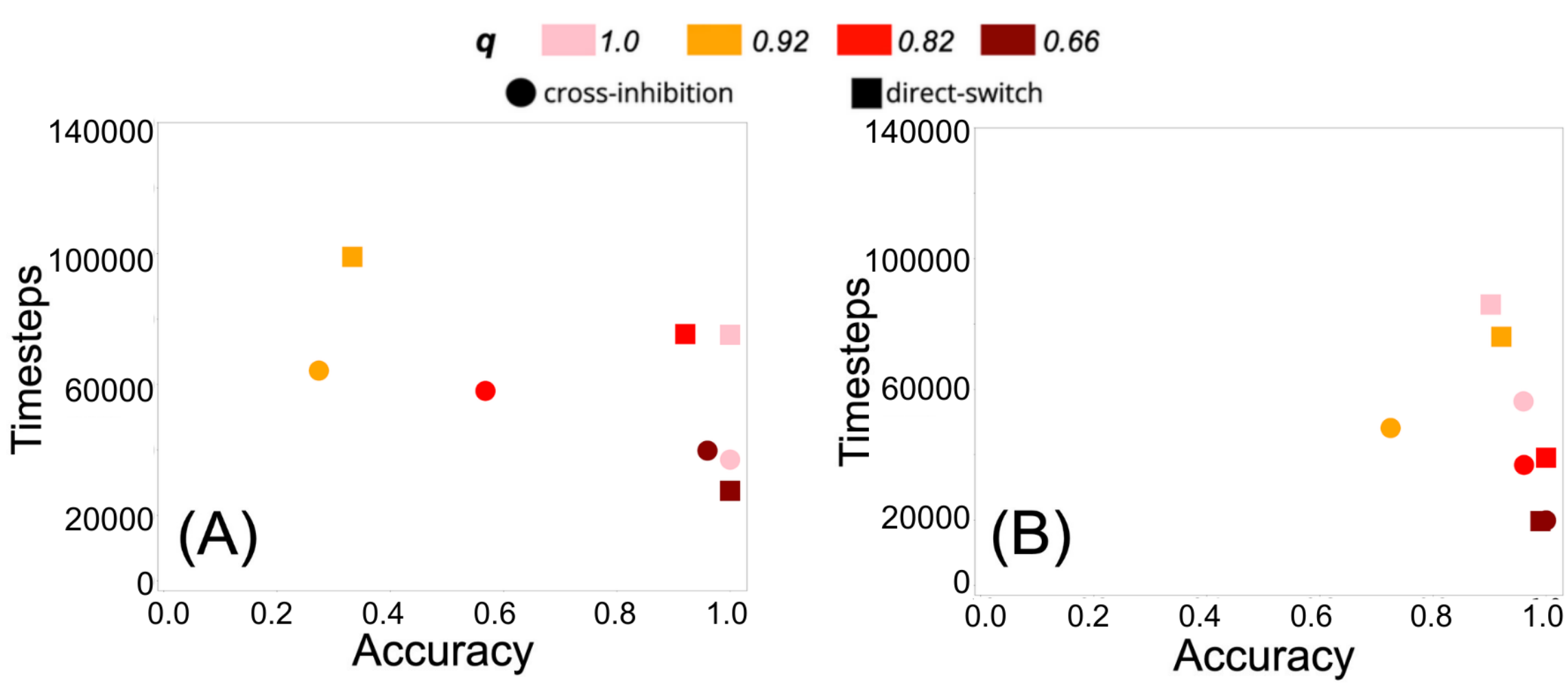

Supplementary Figure 11. (A-B) Speed-accuracy plots from robot simulations showing decision time (y-axis) versus accuracy (x-axis) for cross-inhibition type-2 (circles) and direct-switch (squares). In (A), $\eta_a = 0.1$; in (B), $\eta_a = 0.5$. In both panels, $\eta = 0.05$. Each configuration is evaluated over 50 robot simulations. The robot results are in agreement with the Gillespie heatmaps shown in main text Figure 5.

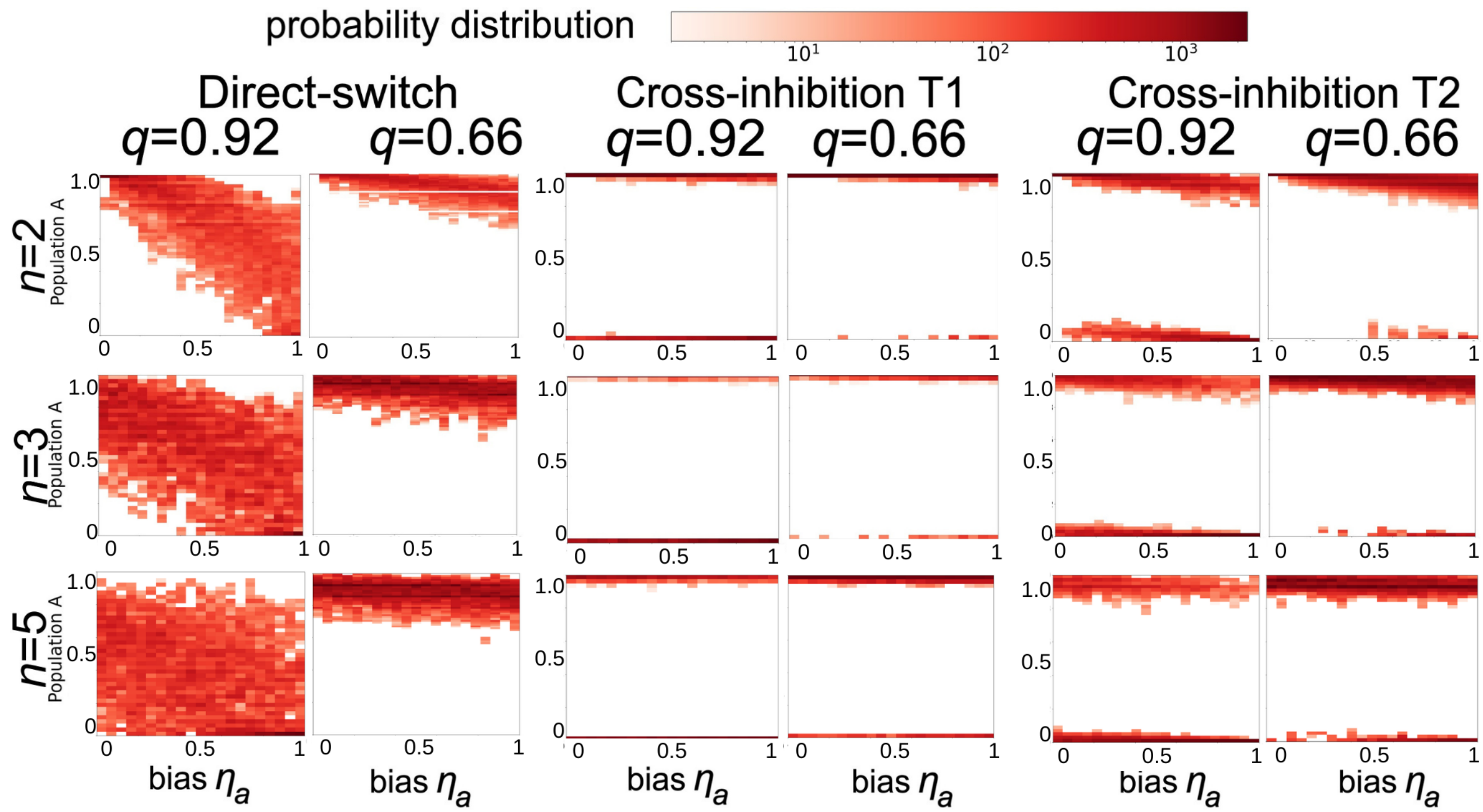

Supplementary Figure 12. Gillespie simulation results are computed in the same manner as in main text Figures 3 and 4, using the same parameter set. We report the steady-state population committed to option A as a function of the self-sourcing bias of option B (η_b) for $\eta = 0.05$. In this formulation, $\eta_b < 0.5$ corresponds to an antagonistic bias, $\eta_b > 0.5$ to a synergistic bias, and $\eta_b = 0.5$ to unbiased asocial dynamics, where the remaining fraction $1 - \eta_b$ is distributed uniformly among the n options. Results are shown for $n = 2, 3, 5$ and for quality ratios $q = 0.92$ and $q = 0.66$, for both the cross-inhibition (type-1 and type 2) and direct-switch mechanisms. The qualitative trends remain consistent across different values of n. When the options are similar ($q = 0.92$), the direct-switch mechanism establishes a majority only within a limited range of η_b, and this majority decreases as the number of options increases. For example, a majority for option A is obtained for $\eta_b < 0.5$ when $n = 2$, whereas this range shrinks to approximately $\eta_b < 0.25$ when $n = 3$. In contrast, when the quality difference is large ($q = 0.66$), the direct-switch mechanism yields a majority for the best option, although this majority can become relatively small for strong antagonistic bias (low η_b) and larger n. For the cross-inhibition mechanism, the system never reaches a deadlock and always breaks symmetry, regardless of the antagonistic bias against option A (i.e., η_b) and the number of options n. However, the inferior option can occasionally be selected; for $n > 2$, the probability of selecting the inferior option occurs at lower values of η_b compared to the $n = 2$ case.

References

1. Doering, G. N. & Pratt, S. C. Symmetry breaking and pivotal individuals during the reunification of ant colonies. *J. Exp. Biol.* **222**, jeb194019 (2019).
2. Franks, N. R., Dornhaus, A., Fitzsimmons, J. P. & Stevens, M. Speed versus accuracy in collective decision making. *Proc. R. Soc. B* **270**, 2457–2463 (2003).
3. Robinson, E. J., Smith, F. D., Sullivan, K. M. & Franks, N. R. Do ants make direct comparisons? *Proc. R. Soc. B* **276**, 2635–2641 (2009).
4. Lichtenberg, E., Imperatriz-Fonseca, V. L. & Nieh, J. C. Behavioral suites mediate group-level foraging dynamics in communities of tropical stingless bees. *Insectes Soc.* **57**, 105–113 (2010).
5. Holway, D. A. & Case, T. J. Effects of colony-level variation on competitive ability in the invasive Argentine ant. *Anim. Behav.* **61**, 1181–1192 (2001).
6. Campo, A. *et al.* Artificial pheromone for path selection by a foraging swarm of robots. *Biol. Cybern.* **103**, 339–352 (2010).
7. Schmickl, T., Hamann, H., Wörn, H. & Crailsheim, K. Two different approaches to a macroscopic model of a bio-inspired robotic swarm. *Robot. Auton. Syst.* **57**, 913–921 (2009).
8. Arvin, F., Turgut, A., Krajník, T. & Yue, S. Investigation of cue-based aggregation in static and dynamic environments with a mobile robot swarm. *Adapt. Behav.* **24**, 102–118 (2016).
9. Campo, A., Garnier, S., Dédriche, O., Zekkri, M. & Dorigo, M. Self-organized discrimination of resources. *PLoS ONE* **6**, e19888 (2010).
10. Amé, J.-M., Halloy, J., Rivault, C., Detrain, C. & Deneubourg, J.-L. Collegial decision making based on social amplification leads to optimal group formation. *Proc. Natl Acad. Sci. USA* **103**, 5835–5840 (2006).
11. Reina, A., Valentini, G., Fernández-Oto, C., Dorigo, M. & Trianni, V. A design pattern for decentralised decision making. *PLoS ONE* **10**, e0140950 (2015).
12. Cai, G. & Lynch, N. A geometry-sensitive quorum sensing algorithm for the best-of-n site selection problem. in *Swarm Intelligence* (eds Dorigo, M. et al.) 1–13 (Springer, Cham, 2022).
13. Cody, J. R., Roundtree, K. A. & Adams, J. A. Human-collective collaborative target selection. *ACM Trans. Hum.-Robot Interact.* **10**, 1–29 (2021).
14. De Masi, G. *et al.* Robot swarm democracy: the importance of informed individuals against zealots. *Swarm Intell.* **15**, 315–338 (2021).
15. Antonic, N., Zakir, R., Dorigo, M. & Reina, A. Collective robustness of heterogeneous decision-makers against stubborn individuals. in *Proc. 23rd Int. Conf. Autonomous Agents and Multiagent Systems (AAMAS 2024)* 68–77 (IFAAMAS, Richland, 2024).
16. Prasetyo, J., De Masi, G. & Ferrante, E. Collective decision making in dynamic environments. *Swarm Intell.* **13**, 217–243 (2019).
17. Ferrante, E. *et al.* A self-adaptive communication strategy for flocking in stationary and non-stationary environments. *Nat. Comput.* **13**, 225–245 (2014).
18. Sion, A., Reina, A., Birattari, M. & Tuci, E. Controlling robot swarm aggregation through a minority of informed robots. in *Swarm Intelligence (ANTS 2022)* (eds Dorigo, M. et al.) 91–103 (Springer, Cham, 2022).
19. Firat, Z., Ferrante, E., Gillet, Y. & Tuci, E. On self-organised aggregation dynamics in swarms of robots with informed robots. *Neural Comput. Appl.* **32**, 13825–13841 (2020).
20. Bartashevich, P. & Mostaghim, S. Benchmarking collective perception: new task difficulty metrics for collective decision-making. in *Progress in Artificial Intelligence* (eds Moura Oliveira, P., Novais, P. & Reis, L. P.) 699–711 (Springer, Cham, 2019).
21. Shan, Q. & Mostaghim, S. Discrete collective estimation in swarm robotics with distributed Bayesian belief sharing. *Swarm Intell.* **15**, 377–402 (2021).
