# Supplementary material for: Bio-inspired decision making in robot swarms under biases
Source: Nat Commun. 2026 Aug 14;17:9774. doi: 10.1038/s41467-026-76408-4 (PMC13572407; doi:10.1038/s41467-026-76408-4)
Supplement: Supplementary file 3 [file 41467_2026_76408_MOESM2_ESM.pdf]

The Description of Additional Supplementary Files

Supplementary Video 1
Description: The main supplementary video summarizing the research

Supplementary Video 2
Description: Robot experiment video for CI, $\boldsymbol{q}$=0.66, $\boldsymbol{\eta}_a$=0.3 and $\boldsymbol{\eta}$=0.05

Supplementary Video 3
Description: Robot experiment video for CI, $\boldsymbol{q}$=0.66, $\boldsymbol{\eta}_a$=0.5 and $\boldsymbol{\eta}$=0.05

Supplementary Video 4
Description: Robot experiment video for CI, $\boldsymbol{q}$=0.92, $\boldsymbol{\eta}_a$=0.3 and $\boldsymbol{\eta}$=0.05

Supplementary Video 5
Description: Robot experiment video for CI, $\boldsymbol{q}$=0.92, $\boldsymbol{\eta}_a$=0.5 and $\boldsymbol{\eta}$=0.05

Supplementary Video 6
Description: Robot experiment video for DS, $\boldsymbol{q}$=0.66, $\boldsymbol{\eta}_a$=0.3 and $\boldsymbol{\eta}$=0.05

Supplementary Video 7
Description: Robot experiment video for DS, $\boldsymbol{q}$=0.66, $\boldsymbol{\eta}_a$=0.5 and $\boldsymbol{\eta}$=0.05

Supplementary Video 8
Description: Robot experiment video for DS, $\boldsymbol{q}$=0.92, $\boldsymbol{\eta}_a$=0.3 and $\boldsymbol{\eta}$=0.05

Supplementary Video 9
Description: Robot experiment video for DS, $\boldsymbol{q}$=0.92, $\boldsymbol{\eta}_a$=0.5 and $\boldsymbol{\eta}$=0.05

Supplementary Video 10
Description: 3D view showing the distinct unstable eigendirections for Fig 7H
